# The CHCHD2-CHCHD10 protein complex is modulated by mitochondrial dysfunction and alters lipid homeostasis in the mouse brain

**DOI:** 10.1038/s41419-025-08030-z

**Published:** 2025-10-06

**Authors:** Jule Gerlach, Paola Pireddu, Xiaoqun Zhang, Simon Wetzel, Mara Mennuni, Dusanka Milenkovic, Hendrik Nolte, Fernanda da Silva Rodrigues, Niclas Branzell, Ibrahim Kaya, Rodolfo Garcia Villegas, Diana Rubalcava-Gracia, David Alsina, Regina Feederle, Per E. Andrén, Thomas Langer, Per Svenningsson, Roberta Filograna

**Affiliations:** 1 Department of Medical Biochemistry and Biophysics, Karolinska Institutet, Stockholm, Sweden; 2 Department of Clinical Neuroscience, Karolinska Institutet, Stockholm, Sweden; 3 Max Planck Institute for Biology of Ageing, Cologne, Germany; 4 Department of Pharmaceutical Biosciences, Spatial Mass Spectrometry, Science for Life Laboratory, Uppsala University, Uppsala, Sweden; 5 Departamento de Biología Molecular y Biotecnología, Instituto de Investigaciones Biomédicas, Universidad Nacional Autónoma de México, Mexico City, México; 6 Core Facility Monoclonal Antibodies, Helmholtz Zentrum München Deutsches Forschungszentrum für Gesundheit und Umwelt (GmbH), Neuherberg, Germany

**Keywords:** Neuroscience, Molecular biology

## Abstract

The highly conserved CHCHD2 and CHCHD10 are small mitochondrial proteins residing in the intermembrane space. Recently, mutations in the genes encoding these proteins have been linked to severe disorders, including Parkinson’s disease and amyotrophic lateral sclerosis. In cultured cells, a small fraction of CHCHD2 and CHCHD10 oligomerize to form a high molecular weight complex of unknown function. Here, we generated a whole-body *Chchd2* knockout mouse to investigate the in vivo role of CHCHD2 and its protein complex. We show that CHCHD2 is crucial for sustaining full motor capacity, normal striatal dopamine levels, and lipid homeostasis in the brain of adult male mice. We also demonstrate that in mouse tissues, CHCHD2 and CHCHD10 exist exclusively as a high molecular weight complex, whose levels are finely tuned under physiological conditions. In response to mitochondrial dysfunction, the abundance and size of the CHCHD2-CHCHD10 complex increase, a mechanism conserved across different tissues. Although the loss of CHCHD2 does not abolish CHCHD10 oligomerization, it enhances cell vulnerability to mitochondrial stress, suggesting that CHCHD2 is protective against mitochondrial damage. Our findings uncover the role of CHCHD2 in preserving tissue homeostasis and provide important insights into the involvement of the CHCHD2-CHCHD10 complex in human diseases.

## Introduction

Mitochondria are essential organelles for bioenergetics, cellular metabolism, and signaling, characterized by a complex architecture with distinct compartments. The mitochondrial intermembrane space houses ~10% of the mammalian mitochondrial proteome [1], including proteins belonging to the coiled-coil-helix-coiled-coil-helix (CHCH) domain family. These proteins are crucial regulators of OXPHOS, lipid homeostasis, mitochondrial ultrastructure and dynamics [2]. Notably, CHCHD3 and CHCHD6, are key components of the mitochondrial contact site and cristae organizing system (MICOS) complex [3]. Genetic variants in CHCH-domain proteins have been implicated in a broad spectrum of human diseases including cancer, obesity, myopathy and neurodegenerative disorders [2].

Mutations in *CHCHD2* have been recently linked to late-onset and juvenile Parkinson’s disease (PD), dementia with Lewy bodies, and sporadic PD [4–7]. Although rare, more than twenty disease-associated *CHCHD2* variants have been reported (see review [8]), primarily autosomal-dominant mutations, suggesting gain-of-function toxicity [9]. However, the identification of nonsense and recessive CHCHD2 mutations supports a pathological mechanism driven by haploinsufficiency [7, 10, 11].

In humans, CHCHD2 is a 151 amino acid protein presenting 58% sequence identity with the family member CHCHD10 [12]. Previous studies performed in fibroblasts and HEK293 cells have shown that CHCHD2 and CHCHD10 are highly abundant as monomers or dimers, but they can also assemble into a high molecular weight complex of ~140–220 kDa [13, 14]. Notably, mutations in *CHCHD10* have been identified in patients with amyotrophic lateral sclerosis and frontotemporal dementia [15, 16], mitochondrial myopathy [17], spinal muscular atrophy [18], and Charcot-Marie-Tooth disease [19].

Extensive research using in vivo and in vitro models has expanded our understanding of CHCHD2 and CHCHD10 but has also produced conflicting outcomes. Initial reports described CHCHD2 and CHCHD10 as MICOS subunits [15, 20], while more recent data argue that CHCHD2 and CHCHD10 mutants affect mitochondrial cristae architecture and only indirectly compromise MICOS stability [21, 22]. The loss of the CHCHD2/CHCHD10 ortholog in flies leads to a reduction in mitochondrial oxygen consumption and alterations in cristae structure [23]. In contrast, CHCHD2 ablation in mice produced inconsistent phenotypes. One report described that 11-month-old *Chchd2* deficient mice do not exhibit motor defects or OXPHOS impairment in the brain [24], whereas another observed that older *Chchd2* knockout mice develop mild motor abnormalities and a subtle loss of midbrain dopamine (DA) neurons [25]. Likewise, *CHCHD10*-deficient mice do not show striking gross and molecular defects [14], suggesting that CHCHD2 and CHCHD10 are functionally redundant and can partially compensate for each other.

Despite the substantial interest in CHCHD2 and CHCHD10 in relation to human diseases, numerous questions remain about the abundance, size, composition, and function of the CHCHD2-CHCHD10 complex in vivo.

In this study, we generated a whole-body *Chchd2* knockout mouse to investigate the role of CHCHD2 in mitochondria. We thoroughly explored the consequences of CHCHD2 loss on motor performance and mitochondrial function. We characterized the CHCHD2-CHCHD10 protein complex in mouse tissues and assessed its significance under conditions of mitochondrial dysfunction. Our findings provide new insights into the in vivo function of CHCHD2 in mammalian mitochondria and underscore its critical role in sustaining brain function and homeostasis.

## Materials and methods

### Ethical statement

All procedures involving animals were conducted following the ethical standards and European, national and institutional guidelines. Protocols were approved by the Stockholm ethical committee and animal work was performed following the guidelines of the Federation of European Laboratory Animal Science Associations (FELASA). For CHCHD2 antibody production, animal experiments were conducted in accordance with the German animal welfare law and performed with permission and adherence to all relevant guidelines and regulations of the district government of Upper Bavaria (Bavaria, Germany; Animal protocol number ROB-55.2Vet-2532.Vet_03-17-68).

### Generation of a monoclonal antibody against CHCHD2

A Wistar rat was immunized with a mixture of 60 µg purified his-tagged mouse CHCHD2 protein in 400 µl PBS, 5 nmol CpG2006 (TIB MOLBIOL, Berlin, Germany), and 400 µl incomplete Freund’s adjuvant. A boost without Freund’s adjuvant was given 12 weeks after primary immunization. Fusion of the myeloma cell line P3X63-Ag8.653 with the rat immune spleen cells was performed 3 days later according to standard procedure [26]. Hybridoma supernatants were screened 10 day later in a flow cytometry assay (iQue, Intellicyt; Sartorius) for binding to CHCHD2 protein captured on beads (3D-Carboxy, PolyAn, Berlin). Antibody binding was analyzed using ForeCyt software (Sartorius). Positive supernatants were further validated by Western blot on mouse wildtype and *Chchd2* knockout tissue. Hybridoma cells of clone CCHD 16D9 (rat IgG2a/k) were subcloned twice by limiting dilution to obtain a stable monoclonal cell line.

### Mouse models

Homozygous mice for a *LoxP*-flanked *Chchd2* allele (*Chchd2*^*loxP/lo*xP^) were crossed to heterozygous β-actin-cre to generate whole-body heterozygous *Chchd2* knockout (*Chchd2*^*+/−*^) mice, which were inter-crossed to obtain homozygous knockout mice (*Chchd2*^*−***/***−*^). Animals were housed in a 12-h light/dark cycle at 21 °C and fed with a standard diet *ad libitum*. Analyses of control and knockout mice were performed at different time points. All mice were on the C57BL/6N background.

Snap frozen tissues and mitochondria isolated from (i) heart and skeletal muscle tissue-specific *Tefm* knockout mice (*Tefm*^*loxP/lo*xP^, +/*Ckmm-cre*) at 8 weeks of age [27], (ii) heart and skeletal muscle tissue-specific *RNAseH1* knockout mice (*RNaseH1* ^*loxP/lo*xP^, +/*Ckmm- cre*) at 24 weeks of age [28] (iii) skeletal muscle tissue-specific *Tfam* knockout mice (*Tfam*^*loxP/lo*xP^, +/*Mlc1f-cre*) at 21 week of age [29], (iv) liver of mice treated with IMT for four weeks [30] were obtained from Prof. Nils-Göran Larsson.

### Behavioral tests

Behavioral analyses in control (*Chchd2*^*+/+*^) and knockout (*Chchd2*^*−/−*^) mice were performed at 6, 9, 12 and 20 months of age. Distinct testing sessions were conducted for female and male animals. Following an acclimation period of at least 30 min in the ventilated experimental room, each test/training session was performed at the same time during the day (9 am–5 pm).

### Open field and rotarod assays

Animal cohorts were tested for spontaneous locomotion in an open field arena using the ActiMot detection system (TSE systems). Free and uninterrupted movements were recorded for 60 min, and spontaneous horizontal/vertical activities and total distance traveled were calculated. Motor coordination was assessed by using a rotarod device (Ugo Basile, Italy). Two training sessions of 90 s at the fixed rotation speed of 4 rpm were conducted a day before to the assessment. On the test day, mice were placed on the rod in acceleration mode (4–40 rpm) for 5 min and the latency to fall off the rod was recorded. Each animal had five trials with a 5-min break in between trials.

### Grip-strength test and Catwalk

Muscle strength in both the fore- and hindlimbs was tested using the grip strength apparatus (BioSeb, FL, USA). Mice were gently held by the tail base and encouraged to grip a metal grid using their forepaws and hind paws. A horizontal force was applied pulling the mice’s tails. The peak force in grams (g) was quantified using a digital force gauge linked to the metal grid. Each animal was tested three times with a 5-min break between each measurement. Gait analysis was performed using the CatWalk device (Noldus, Netherlands). Prior to testing, mice underwent a training phase spanning two days to adapt to the test environment. The CatWalk system tracked gait patterns by an enclosed walkway with a glass plate, illuminated using fluorescent light, and equipped with a high-speed color camera. On the testing day, each mouse was stationed at the entrance of the tunnel, allowing unrestricted movement back and forth along the walkway. The protocol mandated the completion of three successful runs within 10 min with a duration between 0.5 and 7 s and speed variation between 60 and 66%. Mice unable to meet these criteria were excluded from subsequent analysis.

### High-performance liquid chromatography (HPLC)

Sample preparation and HPLC with electrochemical detection (ECD) were done as previously described [31]. Catecholamine content of mouse prefrontal cortex, hippocampus, striatum and cerebellum was measured as previously described [32, 33]. In brief, different brain regions were homogenized in ice-cold perchloric acid, after centrifugation, the clear eluents were subjected to HPLC-ECD. Calibration curves were generated using standard solutions to quantify catecholamine content in the different sections of the mouse brain. The separation of analytes was achieved using a reversed-phase C18 column, and detection was performed at specific voltages in an analytical cell, with chromatograms acquired and analyzed to determine the concentration of various neurotransmitters and metabolites. Chemicals and reagents, including DA, HVA, 3-MT, DOPAC, 5-HT, 5-HIAA, DOPA, EPI, NE, MHPG, 70% perchloric acid, 85% phosphoric acid and sodium bisulfite were purchased from Sigma Aldrich.

### Quantification of TH+ neurons and nerve terminals

Brains were perfused with phosphate-buffered saline (PBS) and postfixed (4% paraformaldehyde) for 48 h before being sectioned at 60 µm using a vibratome (Leica VT1000, Leica Microsystems, Nussloch GmbH, Germany). Sections were washed twice for 5 min in PBS and incubated in blocking buffer (10% bovine serum albumin, 0.3% Triton-X in PBS) for 1 h. Following this blocking step, the sections were incubated overnight at 4 °C with primary antibodies: mouse anti-Tyrosine hydroxylase (TH) (MAB318, Chemicon) diluted in 2.5% bovine serum albumin and 0.3% Triton-X in PBS (1:500 for the striatum and 1:1000 for the substantia nigra). Next, sections were washed three times for 5 min in PBS and incubated at room temperature with fluorescent-conjugated secondary antibody (goat anti-mouse Alexa Fluor-633, A-21052, Invitrogen) for 2 h (1:400). Following three additional 5-min washes in PBS, sections were mounted on coated slides and covered with coverslips using mounting medium with DAPI (ProLong™ Diamond Antifade Mountant, P36966, Invitrogen). Imaging was performed using an LSM800 confocal microscope (Carl Zeiss) with a 20× (Substantia nigra) or 10x (striatum) objective. For confocal imaging, Z-stacks of 15–20 µm thickness were acquired for each captured region. TH positive neuronal cell bodies in the Substantia nigra were quantified using Imaris software, while TH axonal fiber density in the striatum was assessed by measuring mean fluorescence intensity (MFI) using Fiji (ImageJ).

### Autoradiographic detection of DAT

Fresh frozen sections mounted on Superfrost slides were used for autoradiographic detection of dopamine transporter DAT, as previously described [31].

### Sample preparation for MALDI-MSI

Fresh frozen mouse brains from WT (*n* = 9) and CHCHD2 KO (*n* = 7) were cut at a thickness of 12 μm at prefrontal cortex and striatum levels using a cryostat microtome (Leica CM, Leica Microsystems). Tissue sections were thaw-mounted onto conductive indium tin oxide (ITO) glass slides (Bruker Daltonics) and stored at −80 °C. Sections were desiccated at room temperature for 20 min before spray coating of norharmane matrix solution. The slides were scanned on a flatbed scanner (Epson Perfection V500) after the matrix coating. Norharmane matrix solutions were prepared by dissolving and briefly sonicating the norharmane matrix powder (Sigma Aldrich, N6252) in 80% MeOH (7.5 mg/ml) solution in a glass vial. An automated pneumatic sprayer (HTX-Technologies LLC, Chapel Hill, NC, USA) was used, which was combined with a pump (AKTA FPLC P-905 pump, Cytiva, Uppsala, Sweden) to spray heated matrix solution over the tissue sections. Before the experiments, the pump was kept running at 6 μL/min overnight in between the experiments and 70 μL/min for 2 h before each experiment using a 50% acetonitrile pushing solvent to ensure a stable flow of the solvent with isocratic pressure. The matrix solution was sprayed using instrumental parameters of a solvent flow rate of 70 μL/min at isocratic pressure, a nitrogen flow of 6 psi, spray temperature of 60 °C, 15 passes (all horizontal), a nozzle head velocity of 1200 mm/min, and track spacing of 2.0 mm.

### MALDI- FTICR-MSI analysis of lipids

MALDI-MSI experiments for lipid imaging were performed as previously described [34] Briefly, MSI data were collected in dual polarity (both negative and positive ionization modes) on the same tissue sections using a MALDI-FTICR (Solarix XR 7T-2ω, Bruker Daltonics) mass spectrometer equipped with a Smartbeam II 2 kHz laser. The size of the laser was chosen to give a lateral resolution of 100 μm in both polarities with an offset value of 50 μm accompanying the polarity switch. The instrument was tuned for optimal detection of lipid molecules (*m*/*z* 200–2000) in both polarities using the quadrature phase detection (QPD) (2ω) mode. The time-of-flight values were set at 1.0 ms for negative and 0.8 ms for positive ion mode analysis and the transfer optics frequency was kept at 4 MHz for both polarity analyses. The quadrupole isolation *m*/*z* value (Q1 mass) was set at *m*/*z* 220.00 for both polarity analyses. Spectra were collected by summing 100 laser shots per pixel in both polarities. Both methods were calibrated externally with red phosphorus over an appropriate mass range. Ion signals of *m*/*z* 798.540963 (monoisotopic peak of [PC(34:1)+K]^+^) and *m*/*z* 885.549853 (monoisotopic peak of [PI(38:4)-H]^-^) were used for internal calibration for positive and negative polarity analysis, respectively. The data were initially collected in negative polarity and a 5-min-long dummy experiment in the positive ion mode was performed to ensure the instrument performance after polarity switch. The laser power was optimized at the start of each analysis and then held constant during the MALDI-MSI experiment. Any possible bias due to factors such as matrix degradation or variation in mass spectrometer response was minimized by randomized analysis of the tissue sections.

### MALDI-MSI data processing and statistical analysis

MSI data were initially visualized in FlexImaging (v.5.0, Bruker Daltonics). For further analysis, data from WT (*n* = 9) and CHCHD2 KO (*n* = 7) were imported to SCiLS Lab (v. 2024a Pro, Bruker Daltonics) and combined. Brain regions were annotated according to the Allen Mouse Brain Atlas [35]. We utilized the sliding window function of SCiLS Lab to extract root mean square (RMS) normalized average peak area values for 1435 peaks in the negative ion mode and 1229 peaks in positive ion mode separately from each region of interest including the dorsal striatum, ventral striatum and cortex. The combined 2664 peak list in the mass range *m*/*z* 400–2000 was combined into an Excel file for data analysis. Group-wise comparisons were performed using GraphPad Prism 10.2.3 (GraphPad Software, La Jolla, California, USA). For each brain region, peak intensities were tested for normality test, log₂-transformed, and analyzed using multiple *t*-tests. Volcano plots were used to visualize the magnitude and statistical significance of fold changes, and selected peak features were shown in bar graphs using non-log-transformed, RMS-normalized values. Standard multiple testing correction, such as the False Discovery Rate (FDR), was not applied, as many *m*/*z* values do not represent independent lipid species. As a single lipid can generate multiple peaks due to isotopes, adducts, fragments, and harmonics, applying FDR under the assumption of feature independence would result in overcorrection, increasing the risk of false negatives and potentially masking biologically meaningful changes.

### Identification of lipids

A search of the significant *m/z* values from statistical analysis with a 0.01 *m/z* mass tolerance in LIPID MAPS was conducted for both negative and positive polarities including all the ion types. MALDI-tandem MS (MS/MS) was performed on tissues by collecting spectra from brain regions where the target ion was abundant. The resulting product ions were then compared to product ion spectra of standards in the LIPID MAPS database (Nature Lipidomics Gateway, www.lipidmaps.org) and/or previously published data. In cases where sodium and/or potassium adduct ions of the same lipid species were identified, the brain tissue distribution of the adducts ions and [M+H]^+^ or [M-H]^-^ ions were also compared (Table S8) [34].

### Cell lines

Flp-In T-REx HEK293 (HEK293T, RRID: CVCL_U427, female) were grown in DMEM+GlutaMAX culture media (Thermo Fisher, Cat. no. 31966-021) supplemented with 10% fetal bovine serum (Thermo Fisher, Cat. no. 10270-106), 100 U/ml Penicillin / 100 U/ml Streptomycin (Thermo Fisher, GIBCO, Cat. no. 15140122) and maintained at 37 °C and 5% CO_2_. Cells were passaged every 3 to 4 days. Primary mouse embryonic fibroblasts (MEFs) were isolated from embryos at gestational days 13.5 and maintained in culture in the same medium for about 4 passages.

### Assessment of cell growth and viability

Cells were plated in 96-well plates at a density of 1000–2000 cells per well and (10,000 cells) treatments were administered 24 h after seeding. For growth in galactose, cells were cultured in glucose-free DMEM supplemented with 0.9 g/L galactose, 10% (v/v) FBS, 1 mM sodium pyruvate, 1 mM Uridine, 1x Penicillin/Streptomycin for 96 h. For cell viability experiments, cells were treated either with 250 nM rotenone for up to 72 h or with 150 µM actinonin for up to 118 h.

Cell viability was determined using Cell Counting Kit 8 (Sigma, Cat. no. 96992), which selectively stains viable cells.

### Isolation of mitochondria from mouse tissue

Mice were euthanized by cervical dislocation and several tissues, including brain, heart, skeletal muscle and spinal cord, were collected and washed with ice-cold PBS. Mitochondria were isolated using differential centrifugation. Tissues were cut and gently homogenized within in mitochondrial isolation buffer (MIB: 320 mM sucrose, 10 mM Tris–HCl pH 7.4, 1 mM EDTA) supplemented with 1× Complete protease inhibitor cocktail (Roche) and 0,2% of fatty acid-free bovine serum albumin (BSA) using Schuett homgenplus (Schuett Biotec, cat. no. 3.201-011). The tissue homogenate was centrifuged at 1000 × *g* for 10 min at 4 °C to remove the cell debris and nuclei. The supernatant containing mitochondria was transferred into a clean, tube and centrifuged at 10,000 × *g* for 10 min at 4 °C to pellet mitochondria. After an additional wash in MIB without BSA, crude mitochondrial pellets were resuspended in a small volume of MIB without BSA, snap-frozen in liquid nitrogen and stored at –80 °C.

### Isolation of mitochondria from cultured cells

Cultured cells at 80–90% confluency were scraped from two to three 150 mm dishes, washed twice in cold PBS and then centrifuged at 800 × *g* for 5 min at 4 °C. Cell pellet was then resuspended in isolation buffer (CMIB: 20 mM HEPES pH 7.6, 220 mM mannitol, 70 mM sucrose, 1 mM EDTA) supplemented with 1× Complete protease inhibitor cocktail (Roche) and 2 mg/ml of fatty acid-free bovine serum albumin (BSA). To facilitate swelling, the cell suspension was incubated on ice for 15 min. Subsequently, homogenization was performed with 20 strokes by using a manual homogenizer. The cell homogenate was centrifuged at 800 × *g* for 5 min at 4 °C to remove the cell debris. The supernatant was collected into a new tube and centrifuged at 10,000 × *g* for 10 min at 4 °C. The mitochondrial pellet was washed in CMIB without BSA and centrifuged at 10,000 × *g* for 10 min at 4 °C. Mitochondrial extracts were then resuspended in a small volume of CMIB and used for protein quantification. Protein quantification was conducted using a QubitTM Fluorometer (Thermo Fisher, MA, USA).

### Dual COX/SDH enzyme histochemistry

Tissues were frozen in isopentane (15 s) that was previously cooled to −160 °C in liquid nitrogen. Tissues were cryosectioned at –18° to –20 °C using the Thermo Scientific Microm HM560 cryostat (Thermo Scientific, MA, USA) and were placed onto Polysine microscope slides (VWR, PA, USA, Cat. no. 631-0107). COX/SDH staining was performed as previously described [36].

### Biochemical evaluation of respiratory chain function and ATP production

Respiratory chain enzyme activities were measured in isolated mitochondria from different mouse tissues as previously described [37].

### Transmission electron microscopy

Small pieces from the myocardium or quadriceps were fixed in 2% glutaraldehyde and 1% paraformaldehyde in phosphate buffer, at room temperature for 30 min, followed by 24 h at 4 °C. Samples were rinsed and subjected to post-fixation in 2% osmium tetroxide in phosphate buffer at 4 °C for 2 h. The samples were then subjected to stepwise ethanol dehydration followed by acetone/LX-112 infiltration and embedded in LX-112 block. Ultrathin sections were prepared using an EM UC7 ultramicrotome (Leica Microsystems). The ultrathin sections were contrasted with uranyl acetate followed by Reynolds lead citrate and examined using Hitachi HT7700 microscope (Hitachi, Japan). Lipidic structures and sarcomere alterations in TEM images were manually quantified by three independent researchers blinded to the genotype. A total of five mice per genotype were analyzed.

### Western blot analysis

Five to twenty micrograms of isolated mitochondria were resuspended in 4x commercial Laemmli buffer protein and run into Bolt^TM^ 4-12% bis-tris Plus protein gels (Thermo Fisher, MA, USA, Cat. no. NW00120BOX) using MES or MOPS running buffer under denaturing conditions. Proteins were transferred onto nitrocellulose membrane using iBlot™ 2 Transfer Stacks system (Thermo Fisher, MA, USA, Cat. no. IB23001). Immunodetection was performed according to standard techniques using enhanced chemiluminescence Immun-Star HRP Luminol/Enhancer (Bio-Rad) and imaging on an ChemiDoc XRS+ system (Biorad). Specific information on antibodies used in this project are provided in Table S1.

### BN-PAGE and in-gel activity assays

Fifty to hundred micrograms of mitochondria isolated from mouse tissue (liver, skeletal muscle, heart, brain) or MEFs were lysed in solubilization buffer [20 mM Tris–HCl (pH 7.4), 0.1 mM EDTA, 50 mM NaCl and 10% (v/v) glycerol containing either 1% (w/v) DDM or Digitonin 1% and mixed with loading dye (5% Coomassie Brilliant Blue G-250, 150 mM Bis-Tris and 500 mM 6-aminocaproic acid pH 7.0. Blue native–polyacrylamide gel electrophoresis (BN-PAGE) samples were resolved on 4–16% gradient gels (Thermo Fisher, Cat. no. BN1002BO). The inner chamber was filled with Blue Cathode Buffer [[1x NativePAGE Running buffer (20X), Cat. no. BN2001], [1x NativePAGE™ Cathode Buffer Additive (20X) Catalog number: BN2002], H2O] and the outer chamber with Anode Buffer [1x NativePAGE Running buffer (20X), Cat.no. BN2001, 500 mM Bis-Tris /HCl pH 7.0, 10% digitonin or 10% DDM] at 4 °C. BN gels were further subjected to an in-gel catalytic activity assay to measure Complexes I, IV and V. For complex I activity, the BN-PAGE gels were incubated in 2 mM Tris-HCl pH7.4, 0.1 mg/ml NADH (Roche) and 2.5 mg/ml iodonitrozolium for 5–30 min. For complex IV activity, BN-PAGE gels were incubated in 0,05 mM phosphate buffer pH7.4, 25 mg 3.3-´diamidobenzidine tetrahydrochloride (DAB), 50 mg cytochrome c, 3,75 g Sucrose and 1 mg Catalase for 1 h. For complex V activity, BN-PAGE gels were incubated in Complex V activity: BN-PAGE gels were incubated in the following solution 3.76 mg/ml glycine, 5 mM MgCl₂, 1 mg/ml Triton X-100, 0.5 mg/ml lead acetate and 4 mM ATP (pH 8.4). All steps were carried out at room temperature. Coomassie Imperial Blue staining served as loading control.

### Two-dimensional blue native/SDS gel electrophoresis analysis

Strips of the first dimension BN-PAGE gel were incubated for 30 min in 1% SDS and 1% β-mercaptoethanol, and then loaded into onto a 1D precast 12% NuPAGETM, Bis-Tris, 1.0 mm, Mini Prote or (Thermo Fisher, Cat. no. NP0346BOX) to separate the proteins in the second dimension. The experiment proceeded following the Western blot protocol. Subunits of complex II, IV and V were used to map the molecular weight of different protein complexes.

### Quantification of mtDNA copy number

Genomic DNA was isolated from snap-frozen heart and skeletal muscle (quadriceps) using the DNeasy Blood and Tissue Kit (Qiagen), according to the manufacturer’s instructions. Quantification of mtDNA copy number was performed in triplicates using five ng of DNA using TaqMan Universal Master Mix II and TaqMan probes from Life Technologies. The mtDNA levels were assessed using probes against the mitochondrial genes (ND1 and ND4) and nuclear 18S rRNA gene was used as a loading control. (TaqMan Probes are listed in Table S2)

### Immunoprecipitation (IP) and mass spectrometry analysis

Immunoprecipitation experiments were performed using various antibodies coupled with the Dynabeads Protein G system (Thermo Fisher 10004D), according to manufacturer’s instructions. Briefly, for each 1 mg of mitochondria isolated from HEK 293T cells or mouse heart, 50 μL of DynaBeads were employed, and an additional 12 μL were allocated for the preclearing. For optimal antibody binding to the beads, 1 μg of the chosen antibody was incubated with Dynabeads Protein for 1 h at 4 °C. Mitochondria extracts were prepared in lysis buffer (10 mM Tris–HCl pH 7.5, 150 mM NaCl), supplemented with 1% Digitonin and protease inhibitor (PI). After a centrifugation step at 14,000 × *g* at 4 °C for 10 min, the supernatant containing the mitochondrial lysates fractions was pre-cleared for 30 min with non-coated Dynabeads Protein G to reduce non-specific protein binding to the beads. The immunoprecipitation reaction between precleared lysate and Dynabeads Protein G bound with indicated antibodies was performed for 2 h at 4 °C. After extensive washes, one-quarter of the samples were eluted with 1% SDS for 10 min at room temperature and further employed for Western blot analysis. The remaining beads were analyzed by mass spectrometry. Peptide MS/MS spectra of trypsin-digested proteins were detected. Collected raw data was preprocessed by using MaxQuant software without initial normalization to allow the detection of low-abundant proteins [38]. Reviewed Swiss-Prot Proteomes from experimental organisms in FASTQ-format were used as reference proteomes [39]. Perseus software was employed for downstream analysis of collected mass spectrometry data and detected proteins were filtered for mitochondrial subcellular localization using MitoCarta3.0 [40]. For visualization of volcano plots OmicLoupe (Version v1.1.7) was employed [41].

### Complexome profiling

Isolated mitochondria from quadriceps were subjected to complexome profiling proteomics after resolving of protein complexes by 6–16.5% BN-PAGE as extensively described in [42].

In brief, the BN-PAGE gel lanes were segmented into 64 equal-sized bands and each band was sectioned into 1 mm^3^ pieces. These fragments were then processed in an OASIS® HLB μElution Plate, where they underwent de-staining, reduction, alkylation, tryptic digestion, and desalting. The resulting peptides were suspended in 0.1% formic acid and analyzed via mass spectrometry using an Exploris 480 mass spectrometer coupled with an EASY-nLC 1200 UHPLC system. Peptides were separated on a reversed-phase C18 column (Auoroa Frontiers, 15 cm, 1.7 µm, IonOpticks) over a total 30 min gradient run. Mass spectra were acquired in a Top15 data-dependent manner, with fragmentation achieved through HCD. The RF level was set to 45 and the capillary temperature was set to 275 °C. MS1 spectra were acquired at a resolution of 120 K using an AGC (Automatic gain control) target of 1e6. The MS2 spectra were acquired using a resolution of 15 K, a maximum injection time of 23 ms, and an AGC target of 5e5. Raw data were analyzed using MaxQuant version 2.4.2.0, searching against the Uniprot mouse reference proteome (one fast per gene) containing 21,984 protein sequences (downloaded June 2020). The match between runs algorithm was enabled using default settings. Protein quantification was conducted using iBAO intensity. The data were scaled row-wise between 0 and 1 for representation in a heatmap or line diagram using the Instant Clue Software [43].

### RNA extraction and RT- qPCR

Total RNA was isolated from snap-frozen mouse tissues (heart, skeletal muscle and liver) using TRIzol (Thermo Fisher Scientific) and quantified by Nanodrop spectrophotometer. Reverse transcription was performed using the High-Capacity cDNA Reverse Transcription Kit (Applied Biosystems, Life Technologies). qPCR was performed on a QuantStudio 6 (Thermo Fisher Scientific) using the TaqMan Universal Master Mix II with the following TaqMan probes (listed in Table S2) from Life Technologies. β-Actin was used as a loading control.

### Statistical analysis

GraphPad Prism v10 software (version 10.0.3) was used for statistical analyses and graph plotting. All data are presented as means ± SEM and statistical comparisons were performed using unpaired *t*-test. The number of animals or biological replicates (*n*) used for each experiment is indicated in the figure legends. The statistical analysis of MS spectrometry data comparing IP pull-down samples and controls was carried out using both-sided *t*-test. Values of *p* < 0.05 were considered statistically significant.

## Results

### *Chchd2* ablation progressively impairs motor performance in male mice

**Fig. 1:**
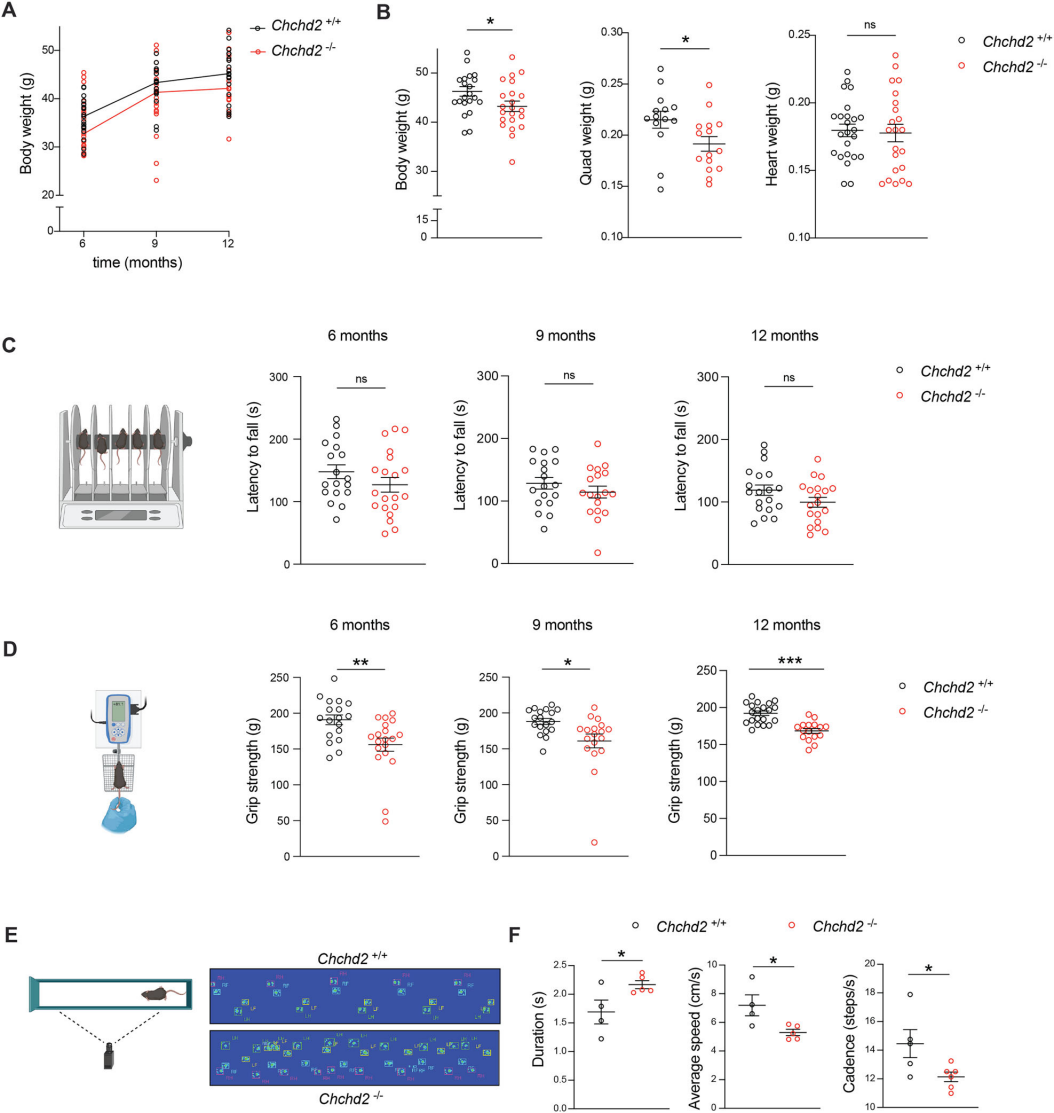
Male mice lacking CHCHD2 exhibit motor defects with age. **A** Body weight (BW) of male *Chchd2* knockout (*Chchd2*^*–/–*^*)* and control (*Chchd2*^*+/+*^*)* mice at 6, 9 and 12 months; *n* > 15 per genotype and time point. **B** BW, quadriceps weight (QW) and heart weight (HW) measured in males at 12 months of age. Data are represented as means ± SEM; *n* > 20 per genotype; *p < 0.05, ns not significant. **C** Motor coordination measured as latency to fall (in s) from the rod in male mice from 6 to 12 months. **D** Muscular strength (in g) measured by grip strength test in male mice from 6 to 12 months. Data are represented as means ± SEM *p < 0.05; *n* > 18 per genotype. ns not significant. **E**, **F** CatWalk gait analysis performed in males at 12 months of age. Representative digitized paw prints are shown in with RF, right front paw, RH, right hind paw, LF, left front paw, and LH, left hind paw. Duration (in s), average speed (cm/s) and cadence (steps/s) to cross the walkway in each step cycle). Data are presented as mean ± SEM. *n* ≥ 4 *p < 0.05.

To study CHCHD2 function, we generated *Chchd2* knockout mice by flanking exons 2–3 with *loxP* sites and crossing them with mice ubiquitously expressing cre-recombinase (Fig. S1A). Homozygous knockout pups (*Chchd2*^*–/–*^*)* were viable and born at Mendelian ratios (Fig. S1B). Western blot analyses confirmed the absence of CHCHD2 in *Chchd2*^*–/–*^ mouse tissues. (Fig. S1C). Although male knockouts were fertile and appeared healthy during early life, they showed reduced body weight starting at 6 months, with a significant reduction by 12 months (Fig. 1A, B). Quadriceps weight was also lower, while heart size and organ-to-body weight ratios remained unaffected (Fig. 1B and Fig. S1D). In contrast, female *Chchd2*^*–/–*^ mice showed no changes in weight even at 20 months (Fig. S1E).

To define whether CHCHD2 loss alters animal motor performance, we tested spontaneous locomotion in open field arenas. Vertical (rearing) and horizontal activities were unaffected in male mice lacking CHCHD2 (Fig. S2A). Rotarod tests revealed minor coordination impairments (Fig. 1C), while grip tests showed a significant decline in muscular strength in male *Chchd2*^*–/–*^ mice between 6 and 12 months (Fig. 1D). Gait analyses performed using CatWalk apparatus showed that knockout mice were slower and presented unstable gait at 12 months (Fig. 1E, F). Female *Chchd2*^*–/–*^ mice did not manifest motor abnormalities at 20 months (Fig. S2B-D).

### CHCHD2 loss affects levels of monoamine neurotransmitters without inducing DA neurodegeneration

**Fig. 2:**
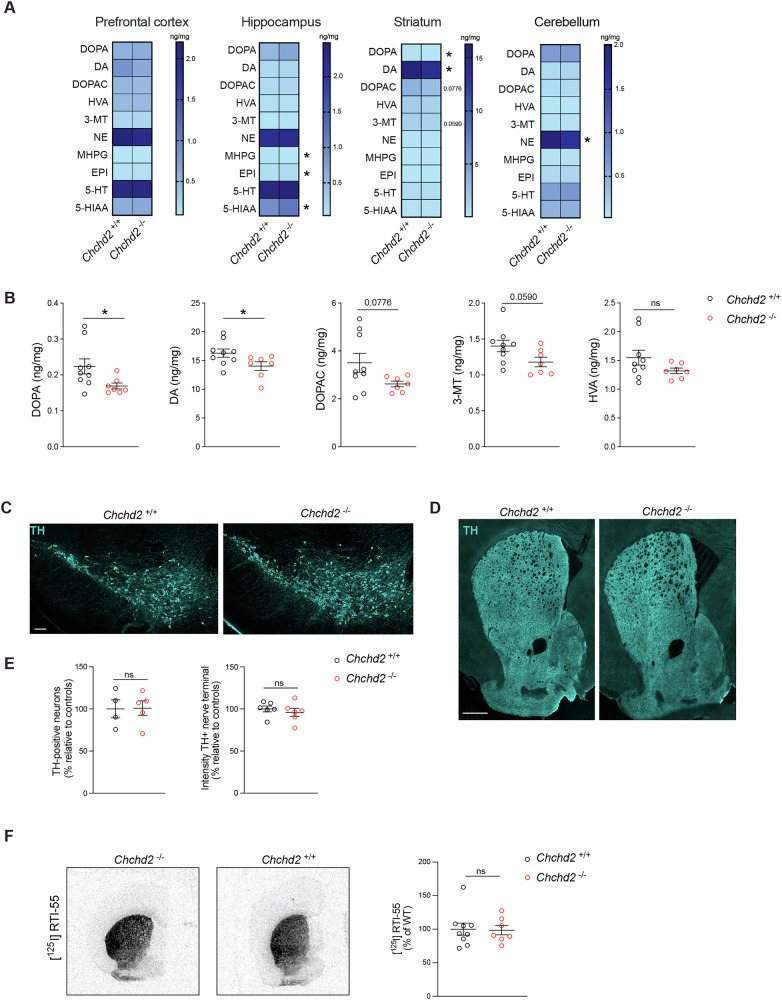
CHCHD2 ablation affects the levels of monoamine neurotransmitters in the brain of male mice. **A** Heatmaps showing the levels (ng/mg) of DA, norepinephrine (NE) and serotonin (5-HT) and their metabolites in the prefrontal cortex, hippocampus, striatum, and cerebellum of *Chchd2*^*–/–*^and *Chchd2*^*+/+*^ male mice at the age of 12 months. **B** Levels (ng/mg) of DOPA, DA, 3,4-Dihydroxyphenylacetic acid (DOPAC), Homovanillic acid (HVA), 3-methoxytyramine (3-MT) in the striatum. Data are represented as means ± SEM; *n* ≥ 7, *p < 0.05, ns not significant. **C**, **D** Representative confocal images of TH-immunoreactivity in sections from midbrain (Scale bars: 100 μm) and striatum (Scale bars: 500 μm). **E** Quantification of TH-positive DA neurons in the midbrain and TH-immunoreactive nerve terminals in the striatum of *Chchd2*^*–/–*^ and *Chchd2*^*+/+*^ mice at the age of 12 months. Data are shown as mean ± SEM. *n* = 3-6. ns not significant. **F** Representative autoradiographs (left panel) and quantification (right panel) of [125I] RTI-55 binding with DAT on striatal sections of *Chchd2*^*–/–*^ and *Chchd2*^*+/+*^ male mice at the age of 12 months. Data are represented as means ± SEM; *n* ≥ 5, ns not significant.

To determine whether the motor defects observed in male *Chchd2*^*–/–*^ mice were due to alterations in monoamine neurotransmitter levels, we measured dopamine (DA), norepinephrine (NE), epinephrine (EPI), serotonin (5-HT), and their metabolites in different brain regions using high-performance liquid chromatography (HPLC) (Fig. 2A). At 12 months, the striatal levels of DA and its precursor DOPA were significantly reduced in knockout mice (Fig. 2B). A similar, though nonsignificant, trend was observed in the DA metabolites 3,4-dihydroxyphenylacetic acid (DOPAC), homovanillic acid (HVA), and 3-methoxytyramine (3-MT) (Fig. 2B). NE and its metabolite 3-methoxy-4-hydroxyphenylglycol (MHPG) were reduced in the cerebellum and hippocampus (Fig. S3A, B), respectively, while EPI and the serotonin metabolite 5-hydroxyindole acetic acid (5-HIAA) were increased in the hippocampus (Fig. S3A). In female mice, HPLC analysis of neurotransmitters in 20-month-old brains revealed no significant differences in the prefrontal cortex, hippocampus, striatum, or cerebellum (Fig. S3C), consistent with the absence of motor defects. However, the ratio of HVA to DA in the prefrontal cortex was higher in *Chchd2*^*–/–*^ females compared to controls (Fig. S3D), which may reflect an increased DA turnover.

Following up on DA alterations in *Chchd2*^*–/–*^ mice, we quantified midbrain DA neurons projecting to the striatum by assessing tyrosine hydroxylase (TH)-positive cells in the *Substantia nigra* (SN) and ventral tegmental area (VTA) using confocal microscopy (Fig. 2C). No significant differences were observed between knockout and control mice at 12 months of age (Fig. 2C). Furthermore, striatal DA innervation, evaluated via TH immunostaining and autoradiographic analysis of the dopamine transporter (DAT), revealed no detectable changes in either TH expression or DAT levels (Fig. 2D–F).

### CHCHD2 loss does not affect OXPHOS capacity but alters tissue morphology

**Fig. 3:**
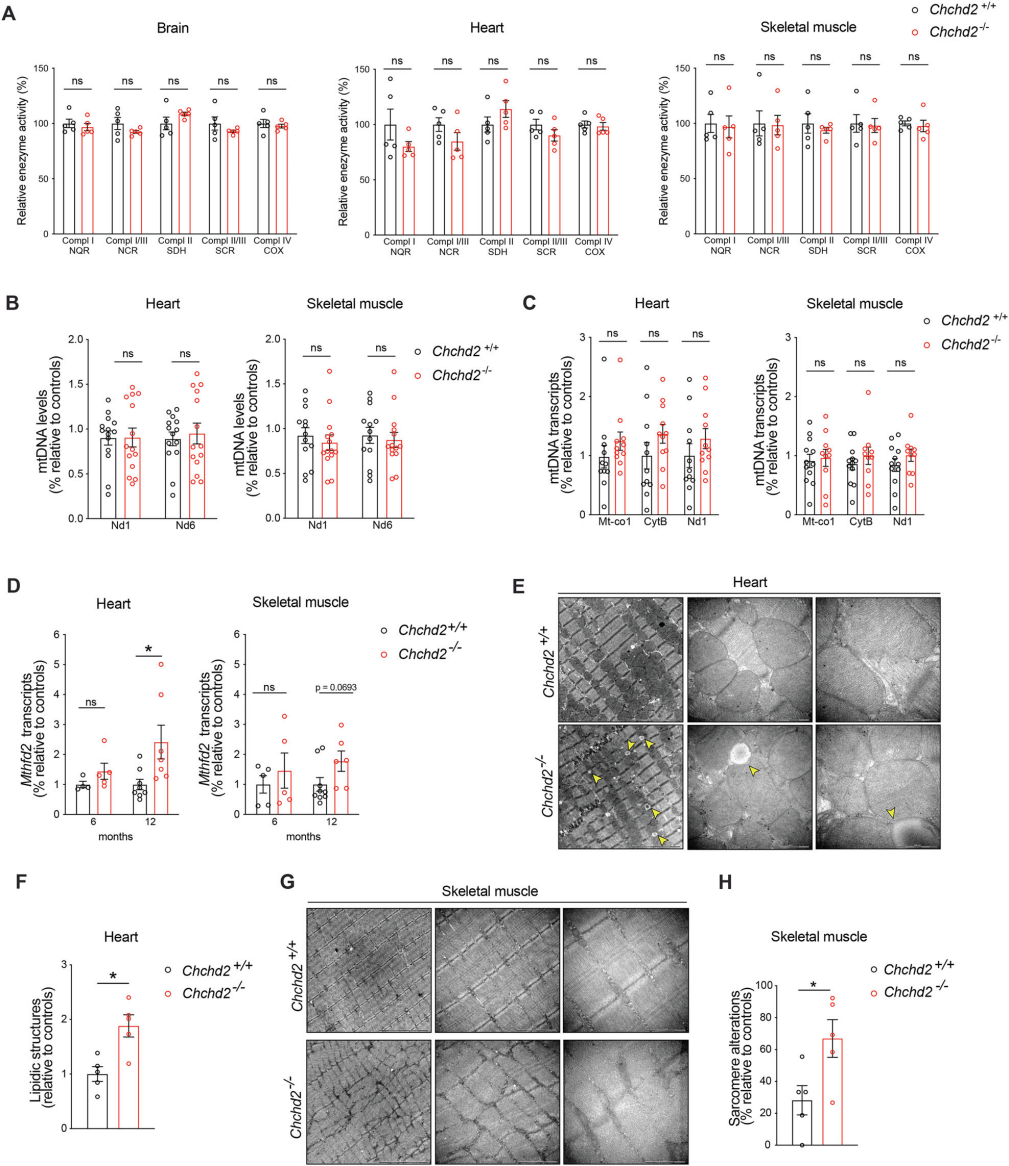
*Chchd2* knockout mice have normal OXPHOS activity but present mild alterations in tissue composition and morphology. **A** Respiratory chain enzyme activity assays of CI, CI+CIII, CII, CII+CIII, and CIV in isolated mitochondria from brain, heart, and skeletal muscle (quadriceps) of *Chchd2*^*–/–*^and *Chchd2*^*+/+*^ male mice at the age of 12 months. Data are represented as means ± SEM.; *n* = 5; ns not significant. Quantification of **B** mtDNA copy number (*Nd1* and *Nd6*/*18S rRNA*) and **C** mtDNA transcripts (*Mt-Co1*, *CytB* and *Nd1*) performed by qPCR in brain, heart, and skeletal muscle. Data are represented as means ± SEM; *n* ≥ 11; ns not significant. **D** *Mthfd2* gene expression measured by qPCR in heart and skeletal muscle of male mice at 6 and 12 months of age. Data are represented as means ± SEM; *n* ≥ 4; *p < 0.05; **E**, **F** Representative electron microscopy images of tissue and mitochondrial morphology (Scale bar: 5, 1 μm and 500 nm) and sample-wise quantification of vacuoles in the heart of male mice at 12 months of age. **G**, **H** Representative electron microscopy images of tissue and mitochondrial morphology (Scale bar: 2 and 1 μm) and sample-wise quantification of sarcomere alterations in the skeletal muscle of male mice at the age of 12 months. Data are represented as means ± SEM; *n* = 5; *p < 0.05.

Next, we examined the effects of CHCHD2 ablation on mitochondrial function across different tissues. At one year of age, *Chchd2*^*–/–*^ mice displayed mostly normal steady-state levels of OXPHOS subunits, with only minor reductions in mitochondrially encoded cytochrome c oxidase I (MT-CO1) in the brain and skeletal muscle (Fig. S4A). In-gel activity assays revealed no changes in complex IV or V (Fig. S4B). Similarly, spectrophotometric analysis of respiratory chain complexes in isolated mitochondria showed normal activities in the brain, heart, and skeletal muscle (Fig. 3A). However, histochemical staining of complex IV (cytochrome c oxidase, COX) and succinate dehydrogenase (SDH) in the colon, a replicative tissue prone to acquire age-associated mitochondrial damage [44], revealed an accumulation of COX-deficient crypts (blue cells) in 12-month-old *Chchd2*^*–/–*^ mice (Fig. S4C).

Given that mutations in *CHCHD10* were reported to cause an increase of mtDNA deletions and a reduction in mtDNA levels in human and mouse tissues [9, 15, 45], we measured mtDNA copy number and mtDNA transcripts in heart and skeletal muscle by qPCR. At one year of age, no changes were observed between control and knockout mice (Fig. 3B, C), suggesting CHCHD2 is not required for mtDNA maintenance or expression. However, *Chchd2*^*–/–*^ mice exhibited upregulation of the *Mthfd2* gene, which encodes a key enzyme involved in mitochondrial one-carbon metabolism and is an early marker of mitochondrial dysfunction [46]. Although modest, this upregulation was progressive between 6 and 12 months, ranging from a 1.4- to 2.5-fold increase in the heart and a 1.4- to 1.8-fold increase in skeletal muscle (Fig. 3D). Notably, this occurred without activation of the mitochondrial integrated stress response (ISR), as indicated by the absence of eIF2α phosphorylation (Fig. S4D) and unchanged transcripts levels of ISR target genes *Atf4*, *Atf5*, *Chop*, and *Fgf21* (Fig. S4E).

To assess tissue morphology and mitochondrial cristae organization, we performed electron microscopy on one-year-old mouse tissues. A higher content of circular vacuoles resembling lipid droplets was observed in the hearts of *Chchd2* knockouts (Fig. 3E, F), similar to those previously reported in muscles of mice expressing pathological CHCHD10 mutants [22] and in a myopathy patient carrying CHCHD10 mutations [45]. In the quadriceps femoris of *Chchd2*^*–/–*^ mice, sarcomere organization was altered, with irregular myofibril and z-disc arrangements (Fig. 3G, H). Despite these changes, mitochondrial ultrastructure and cristae organization remained intact in the heart and skeletal muscle (Fig. 3G, H).

### CHCHD2 loss alters lipid homeostasis in the cortex and dorsal striatum

**Fig. 4:**
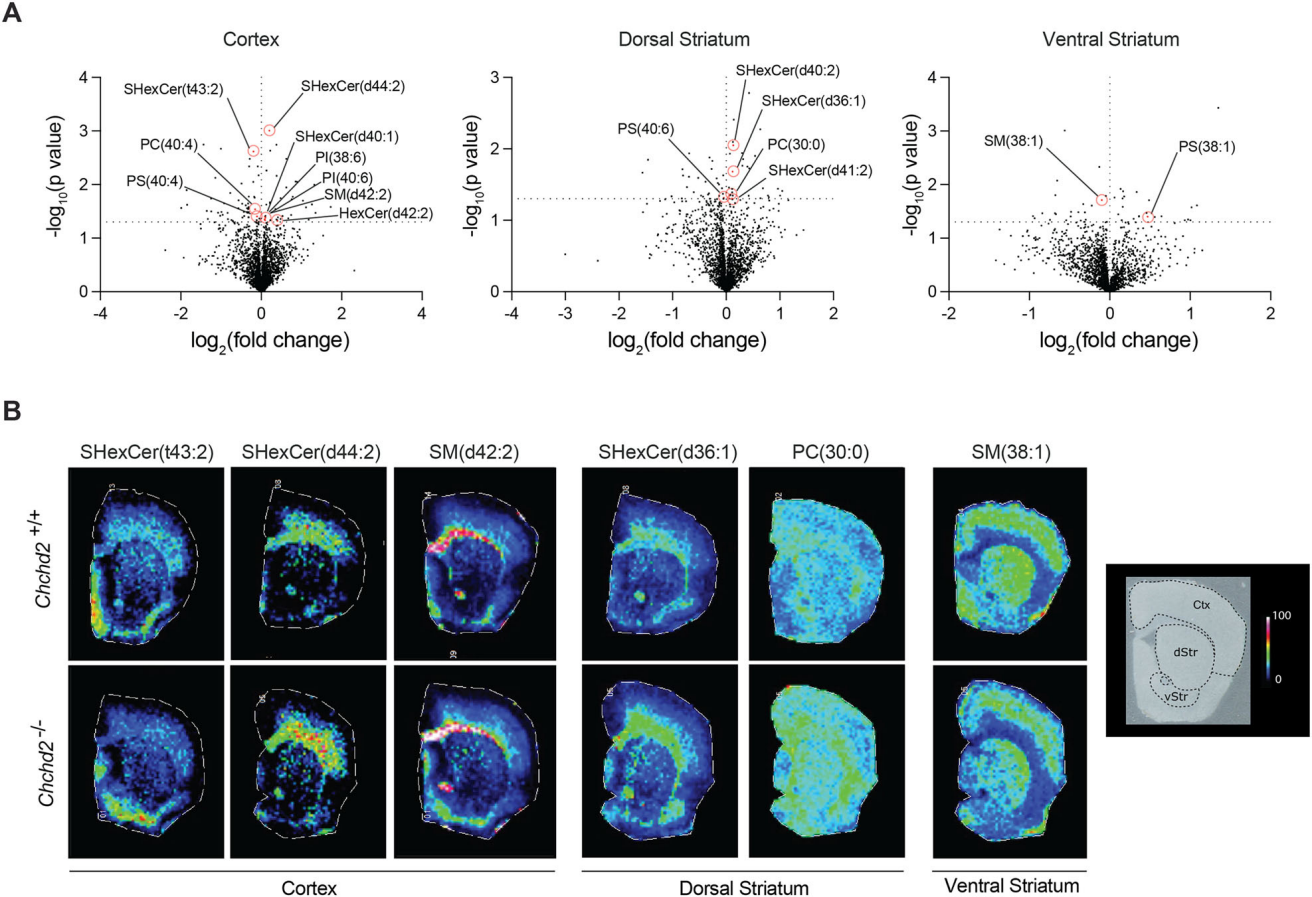
Male mice lacking CHCHD2 exhibit regional alterations in lipid content in the brain. **A** Volcano plots showing log₂(fold changes) and −log₁₀(*p*-values) of all detected *m*/*z* values in the cortex, dorsal striatum, and ventral striatum of *Chchd2*^*–/–*^ (*n* = 7) and *Chchd2*^*+/+*^ (*n* = 9) male mice at 12 months of age. Each brain region was analyzed independently. Peak intensities were normalized, log₂-transformed, and assessed using multiple *t*-tests. Identified regulated lipids are highlighted with the monoisotopic peaks of their corresponding ions, indicated by red dashed lines. **B** Representative ion images of SHexCers (sulfohexosylceramides), HexCers (hexosylceramides), SMs (sphingomyelins), and PCs (Phosphatidylcholines) peaks in the cortex, dorsal and ventral striatum of *Chchd2*^*–/–*^and *Chchd2*^*+/+*^ male mice at the age of 12 months. The lateral resolution of the MALDI-FITCR-MS ion images is 100 µm, and all ion images were RMS-normalized.

The identification of lipid vacuoles in the heart of *Chchd2*^*–/–*^ mice prompted us to examine lipids in the brain. As lipids constitute ~50% of the brain dry mass [47] and are critical for its structure and function, we performed an untargeted spatial lipidome analysis using MALDI-FTICR-MSI [31, 34]. This analysis revealed significant lipid alterations in the cortex and striatum of 12-month-old *Chchd2*-deficient mice (Fig. 4A, B, Fig. S5A and Table S8). Non-hydroxylated sphingolipids (d=nonhydroxylated), such as sulfohexosylceramides (SHexCers), hexosylceramides (HexCers) and sphingomyelins (SMs) were increased in dorsal striatum and cortex, while the hydroxylated SHexCers (t=hydroxylated) were decreased in the cortex (Fig. S5A). Similar changes were recently observed in the brain of a non-human primate PD model generated using 1-methyl-4-phenyl-1,2,3,6-tetrahydropyridine (MPTP) [34]. Additional alterations were detected in phospholipids essential for membrane integrity, including mitochondrial membranes. The polyunsaturated fatty acid-containing phosphatidylinositol, PI (40:6) and PI (38:6) were higher in the cortex, whereas the phosphatidylserines PS (40:4) and PS (40:6) were decreased in the cortex and dorsal striatum, respectively. The levels of the phosphatidylcholine species PC (40:4) and PC (30:0) were also affected in these two brain regions (Fig. S5A). Overall, CHCHD2 ablation causes alterations in lipid homeostasis in different brain regions, including the dorsal striatum, which is particularly affected in PD patients.

### In vivo CHCHD2 and CHCHD10 are fully assembled into a large protein complex

**Fig. 5:**
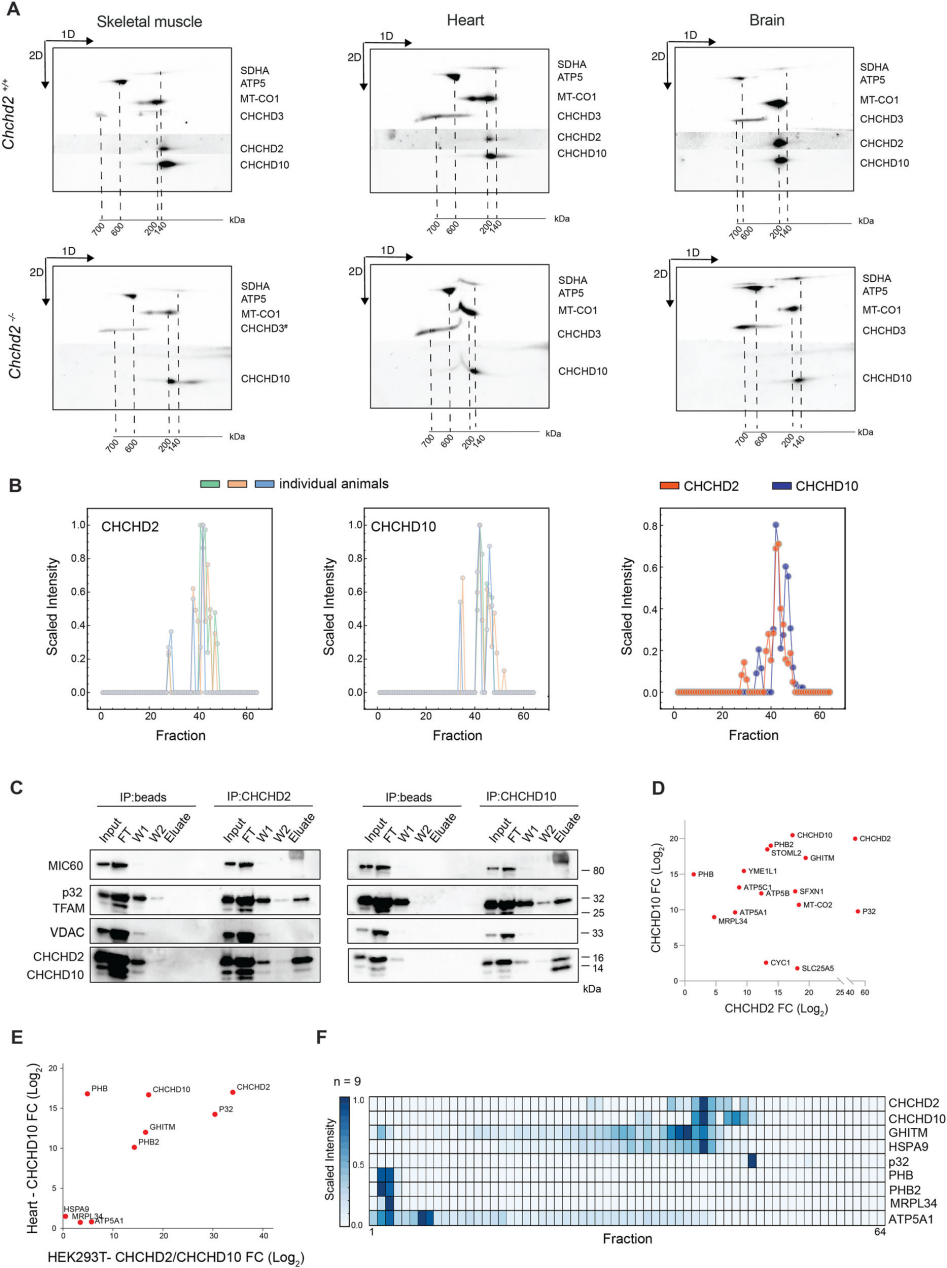
In vivo CHCHD2 and CHCHD10 are fully assembled into a large complex. **A** 2D-PAGE analyses of mitochondria isolated from skeletal muscle, heart and brain of *Chchd2*^–/–^ and *Chchd2*^+/+^ male mice at the age of 12 months. Mitochondria were solubilized using 1% (w/v) DDM. The position of SDHA, ATP5, MT- CO1, and CHCHD3 corresponds to the size of their different protein complexes (in kDa). The molecular weights of monomeric CHCHD2 and CHCHD10 are 15.5 and 14.1 kDa, respectively. **B** Migration profiles of CHCHD2 and CHCHD10 obtained from complexome profiling analysis of mitochondria isolated from skeletal muscle of *Chchd2*^*+/+*^ mice at the age of 12 months. *n* = 3. **C** Representative Western blot analysis of CHCHD2 and CHCHD10 IP experiments using mitochondria isolated from HEK293T cells (*n* = 4–5 IP and control samples). The blots show the presence of CHCHD2 and CHCHD10 in the antibody-bound (ab), flow-through (FT), and wash (W) fractions. **D** Interactome plot of proteins identified by mass spectrometry following CHCHD2 and CHCHD10 IP in HEK293T mitochondria. Only proteins with significant enrichment (*p* < 0.05) are shown. Axes represent the log₂ fold change (Log₂FC) of each protein between IP samples and control inputs. **E** Interactome plot combining in vitro (HEK293T) and in vivo (mouse heart) CHCHD2 and CHCHD10 IP experiments. Only significant hits (*p* < 0.05) are displayed. Axes represent average Log₂FC (Mean_D2_D10) from HEK293T samples and the normalized Log₂FC from CHCHD10 IP in mouse heart tissue. **F** Heatmap from complexome profiling of mitochondria isolated from skeletal muscle (*n* = 3), depicting the relative abundances of proteins identified in panel **E**.

In human cell lines, CHCHD2 is highly abundant as a monomer and dimer, though a smaller fraction can form a large complex with CHCHD10 [13, 14]. To assess the abundance and the size of this protein complex in vivo, we performed two-dimensional gel electrophoresis (2D-PAGE) analyses in mitochondria isolated from brain, heart and skeletal muscle of *Chchd2*^*+/+*^ and *Chchd2*^*–/–*^ mice under mild (digitonin) (Fig. S6A) or stringent (DDM) detergent solubilization conditions (Fig. 5A). In control tissues, CHCHD2 and CHCHD10 co-migrated as a high molecular complex of ~140 kDa, while no monomers or dimers were found (Fig. 5A). In the absence of CHCHD2, CHCHD10 could still oligomerize but formed a less abundant, possibly unstable complex (Fig. 5A). Importantly, neither CHCHD2 nor CHCHD10 co-migrated with CHCHD3, indicating that these proteins are not component of the MICOS. To ensure that the differences in CHCHD2 and CHCHD10 oligomerization observed in tissues were not caused by experimental conditions, we performed 2D-PAGE using mitochondria isolated from HEK293T cells and MEFs (Fig. S6B, C). Consistent with earlier studies [13, 14], CHCHD2 and CHCHD10 were mainly found at low molecular weight, presumably as monomers or dimers, although a minor portion of CHCHD2 migrated at higher molecular weight.

To characterize the CHCHD2-CHCHD10 complex at higher resolution, we employed complexome profiling proteomics of skeletal muscle mitochondria resolved by BN-PAGE (Fig. S6D). Electrophoretic migration profiles showed a striking comigration between CHCHD2 and CHCHD10 from 100 to 140 kDa (fractions 28-45) (Fig. 5B). In line with our 2D-PAGE data, CHCHD2 and CHCHD10 were not detected in low molecular weight fractions (50 to 64), excluding their existence as monomers or dimers in vivo (Fig. 5B). The migration profile of CHCHD10 was not significantly affected in *Chchd2*^*–/–*^ mice (Fig. S6E), validating that CHCHD10 is still able to oligomerize without CHCHD2. Notably, MICOS stability and assembly were unchanged upon CHCHD2 loss (Fig. S6F).

Next, we investigated the CHCHD2-CHCHD10 complex interactome by co-immunoprecipitation (co-IP) of the endogenous proteins using mitochondria isolated from HEK293T cells. Western blot analysis confirmed the strong physical interaction between CHCHD2 and CHCHD10 (Fig. 5C). The previously reported interacting partner p32 (C1QBP) [14, 48] was also found in CHCHD2 and CHCHD10 eluates (Fig. 5C). Label-free quantitative mass spectrometry was applied to the pulldown eluates to determine additional interacting partners (Fig. S6G, H and Table S3, S4). This analysis revealed that CHCHD2 and CHCHD10 interactomes largely overlapped (Fig. 5D and Table S5). The stomatin-like protein 2 (SLP2) and iAAA protease YME1L were highly enriched in both CHCHD2 and CHCHD10 pulldowns (Fig. 5D), whereas the rhomboid protease PARL was specific to CHCHD10 (Fig. S6H). In line with our results, the SLP2-PARL-YME1L protease complex, known as SPY, was found to interact with CHCHD2 [49]. Furthermore, both prohibitin (PHB) complex subunits, PHB1 and PHB2, were co-immunoprecipitated with CHCHD2 and CHCHD10. Interestingly, a recent study has shown that CHCHD10 together with the protease SLP2 controls the stability of the PHB complex [21].

To distinguish core components of the CHCHD2-CHCHD10 complex from transient interactors, we characterized the in vivo CHCHD10 interactome using mitochondria isolated from adult wild-type mouse hearts, applying the same co-immunoprecipitation workflow as in HEK293T cells (Fig. S6I, J, and Table S6). Technical limitations with available antibodies precluded a parallel analysis of the in vivo CHCHD2. Comparison of in vitro and in vivo datasets (Fig. 5E) identified nine proteins consistently enriched in both CHCHD2 and CHCHD10 interactomes from HEK293T cells and in the CHCHD10 interactome from mouse heart (Table S7). To determine whether these shared interactors are stable components of the complex, we surveyed our complexome profiling datasets and generated a heatmap of their relative abundance across fractions (Fig. 5F). None co-migrated precisely with CHCHD2 and CHCHD10, suggesting they associate only transiently, and that the core complex consists solely of CHCHD2 and CHCHD10.

### The levels and the size of the CHCHD2-CHCHD10 complex are regulated by mitochondrial dysfunction

**Fig. 6:**
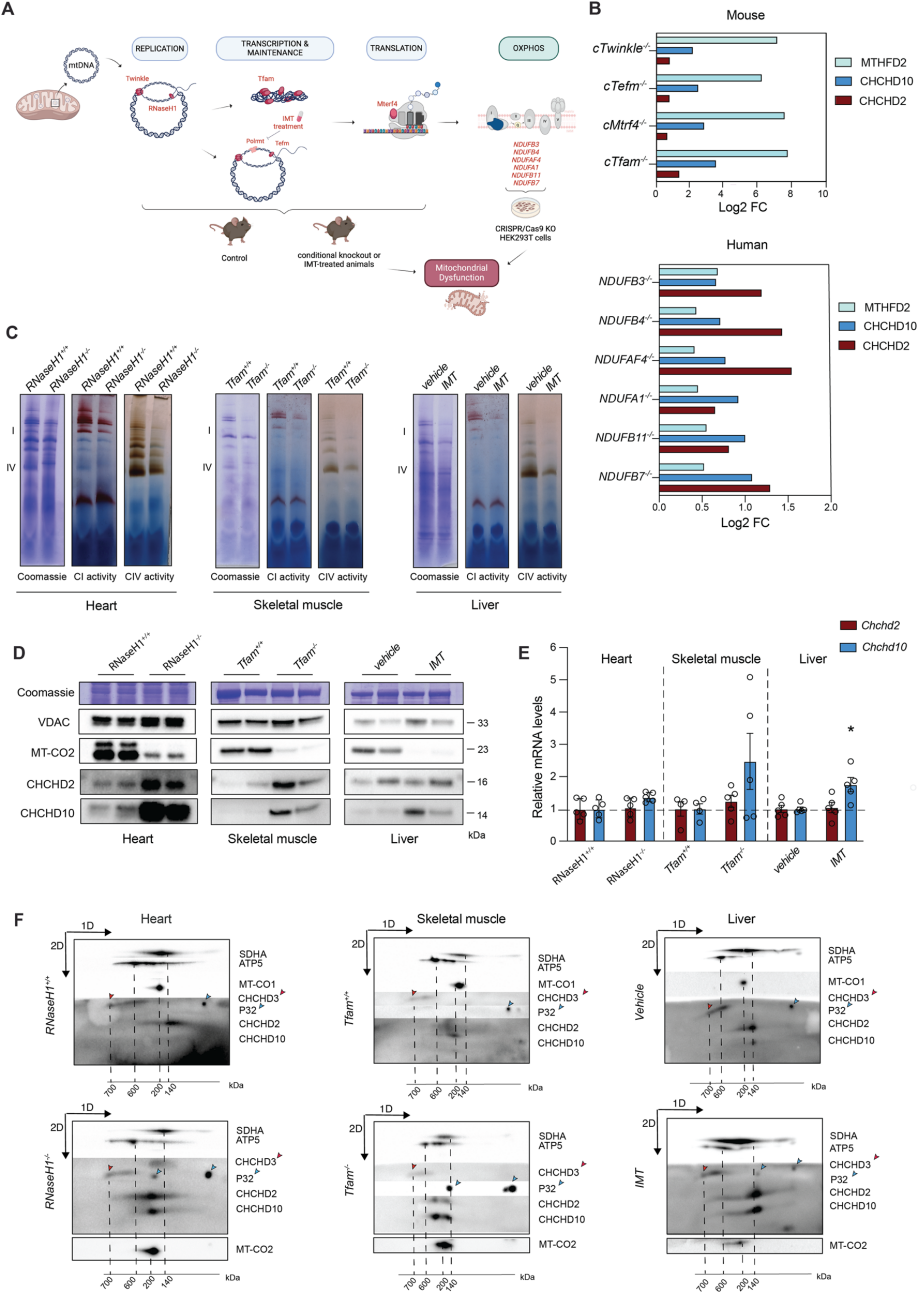
The levels and the size of the CHCHD2-CHCHD10 protein complex are modulated in response to mitochondrial damage. **A** Schematic representation of models with mitochondrial dysfunction. The image was created with BioRender.com. **B** Levels of MTHFD2, CHCHD2, and CHCHD10 (Log_2_FC) obtained using publicly available proteomic datasets from tissue-specific knockout mouse strains with impaired mtDNA gene expression (*Twinkle, Tefm, Mterf4* and *Tfam* knockout mice) and in HEK293T cell lines lacking complex I accessory subunits. **C** Complex I and IV in-gel activities performed on mitochondria isolated from heart tissue of *RNaseH1* mice at 24 weeks of age, skeletal muscle (gastrocnemius) of *Tfam* mice at 5 months of age and liver of IMT treated mice for four weeks and respective controls. BN-PAGE were stained with Coomassie or incubated with substrates for detecting the in-gel activity of the indicated OXPHOS complexes. **D** Western Blot analysis of CHCHCD2 and CHCHD10 steady-state levels in mitochondria isolated from tissues of three different mouse strains affected by mitochondrial dysfunction in heart, skeletal muscle and liver, respectively. VDAC and Coomassie staining were used as loading controls. **E** *Chchd2* and *Chchd10* mRNA levels measured by qPCR from RNA isolated from mouse tissues with mitochondrial dysfunction. Data are represented as mean ± SEM. *n* = 5 mice for each genotype, *p < 0.05. **F** 2D-PAGE of mitochondria isolated from skeletal muscle of from heart tissue of *RNaseH1* mice at 24 weeks of age, skeletal muscle (gastrocnemius) of *Tfam* mice at 5 months of age and liver of IMT treated mice for four weeks and respective controls Mitochondria were solubilized using 1% (w/v) DDM. The position of SDHA, ATP5, MT-CO1, MT-CO2 (used in models of mitochondrial dysfunction) and CHCHD3 (indicated by a red arrow) corresponds to the size of their different protein complexes (in kDa). P32 is indicated by blue arrows.

CHCHD2 and CHCHD10 accumulate in mitochondria under mitochondrial stress conditions [23, 50], which suggests that their expression or stability is influenced by mitochondrial damage and cellular metabolism. To explore this hypothesis, we analyzed CHCHD2 and CHCHD10 protein levels in a variety of in vivo and in vitro models of severe mitochondrial dysfunction (Fig. 6A). Using publicly available proteomic datasets, we examined hearts from conditional knockout mice with disrupted mtDNA gene expression caused by the loss of the mitochondrial DNA helicase (Twinkle), the mitochondrial transcription factor A (Tfam), the mitochondrial transcription elongation factor (Tefm), and the mitochondrial transcription termination factor 4 (Mterf4) [27, 46]. In late-stage disease, when MTHFD2 levels increased dramatically (70- to 215-fold), CHCHD2 and CHCHD10 were upregulated 1.5- to 2.5-fold and 4- to 10-fold, respectively (Fig. 6B). Similar upregulation was observed in proteomic analyses of human cells lacking complex I accessory subunits [51](Fig. 6B). These findings indicate that CHCHD2 and CHCHD10 increase may be a conserved response to mitochondrial damage across species and tissues. Hence, we experimentally examined different tissues from four mouse models with defective mitochondria at their respective endpoints. We analyzed the heart of 24-week-old *Ribonuclease H*1 (*RNaseH1*) knockouts [28], the skeletal muscle of 21-week-old *Tfam* knockout mice [29], and the liver of wild-type mice treated with the inhibitor of mitochondrial transcription (IMT) for four weeks [30]. As a positive control, we included the heart of the previously analyzed 8-week-old *Tefm* knockout model [27]. We determined the magnitude of mitochondrial impairment by measuring the levels of OXPHOS subunits (Fig. S7A) and complex I and complex IV activities (Figs. 6C and S7B), which demonstrated a dramatic reduction in OXPHOS capacity across models. We additionally measured *Mthfd2 mRNA*, which is transcriptionally regulated and correlates with the severity and progression of mitochondrial defects [52]. We found that *Mthfd2* was strongly upregulated (~80 to 150-fold) in the heart and skeletal muscle of mice lacking RNAseH1, TEFM and TFAM (Fig. S7C), in line with profound mitochondrial damage leading to lethality [46]. In the liver of IMT-treated mice, the increase in *Mthfd2* transcript was much milder (~5-fold) (Fig. S7C), consistent with no effects on animal survival and organ function [30]. We assessed CHCHD2 and CHCHD10 levels in isolated mitochondria (Figs. 6D and S7D) and found that CHCHD10 protein levels were markedly increased in heart and skeletal muscle of mice lacking RNaseH1, TEFM and TFAM, whereas only moderately increased in the liver of IMT-treated mice (Figs. 6D and S7D). Although less pronounced, CHCHD2 was upregulated in all the models and tissues (Figs. 6D and S7D). Next, we studied whether this upregulation was due to changes in gene expression (Figs. 6E and S7E). We found that *Chchd2* transcript levels were unchanged across all tissues and models. In contrast, *Chchd10* mRNA was mildly upregulated in the heart of *Tefm* knockout mice (~2.5-fold) and the liver of IMT-treated mice (~1.5-fold) (Figs. 6E and S7E). A similar trend was observed in the heart of *RNaseH1* knockout mice and the skeletal muscle of *Tfam* knockout mice (Fig. 6E). However, the upregulation of the *Chchd10* transcript was not as pronounced as the increase in protein levels.

To determine whether mitochondrial impairment affects the formation of the CHCHD2-CHCHD10 complex, we employed 2D-PAGE on mitochondria isolated from the heart, skeletal muscle, and liver of each mouse strain (Figs. 6F and S7F). Consistent with our previous results, CHCHD2 and CHCHD10 run as a complex of ~140 kDa in healthy mitochondria. In defective mitochondria, the abundance of the CHCHD2-CHCHD10 complex was markedly increased without the accumulation of any smaller forms. Importantly, the CHCHD2-CHCHD10 complex presented a notable shift towards higher molecular weight upon mitochondrial defects in the heart, skeletal muscle and liver (Figs. 6F and S7). Surprisingly, mitochondrial dysfunction also affected the migration pattern of p32, which partially co-migrated with CHCHD2 and CHCHD10.

### CHCHD2 upregulation protects against mitochondrial damage

**Fig. 7:**
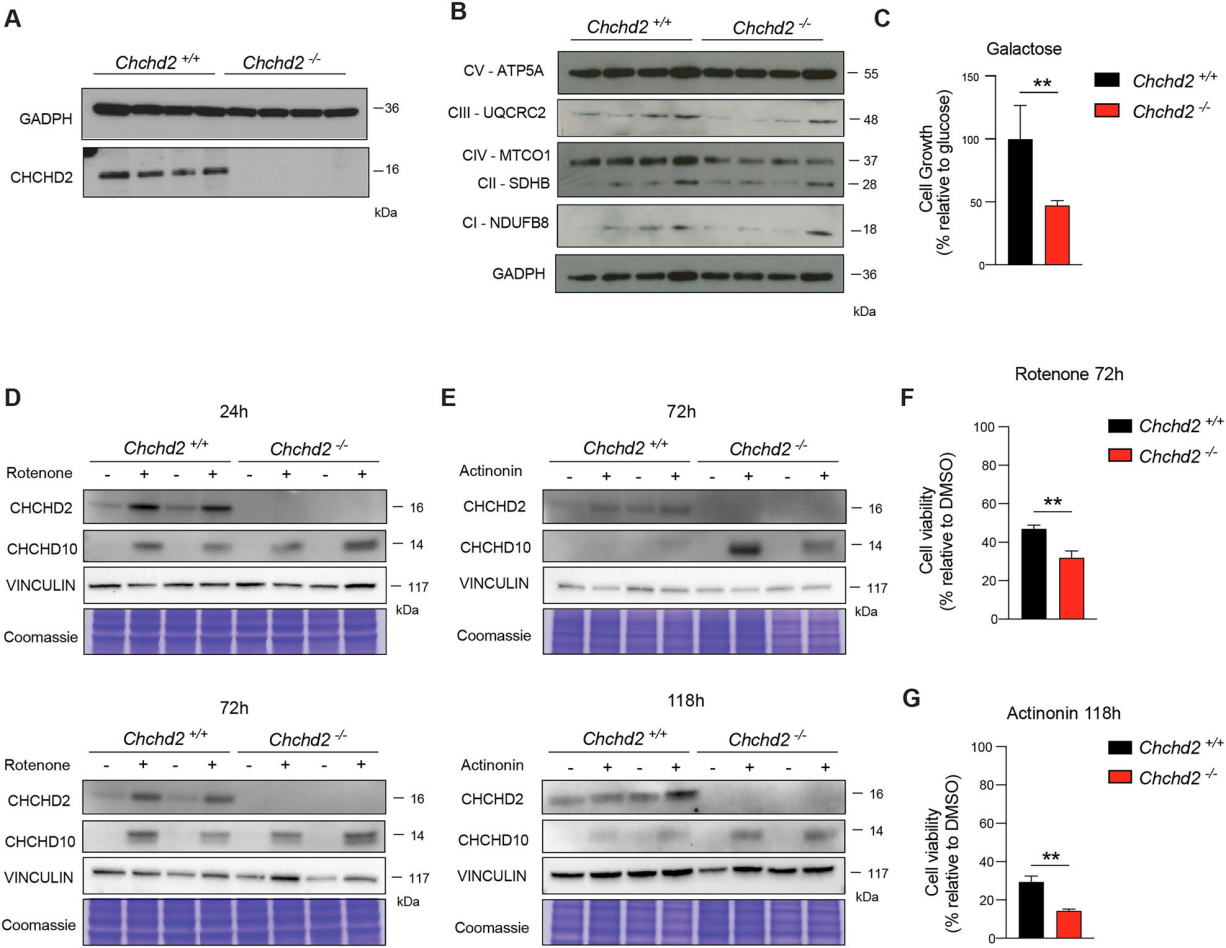
CHCHD2 is required to sustain cell viability under mitochondrial stress conditions. **A**, **B** Western blot analyses of steady-state levels of CHCHD2 and OXPHOS subunits in total extracts from primary *Chchd2*^*–/–*^ and *Chchd2*^*+/+*^ MEFs (*n* = 4 cell lines per genotype). GADPH was used as a loading control. **C** Analysis of cell growth in medium containing 0.9 g/l galactose. Data are expressed as % relative to the cell growth in medium containing glucose and represented as mean ± SEM (*n* = 4 cell lines per genotype); **p < 0.01. **D**, **E** Representative Western Blot analysis of CHCHCD2 and CHCHD10 steady-state levels in total extract from primary *Chchd2*^*–/–*^ and *Chchd2*^*+/+*^ MEFs treated with 250 nM rotenone for 24 and 72 h and 150 nM actinonin for 72 and 118 h. VINCULIN and Coomassie were used as loading controls. **F**, **G** Cell viability performed in primary *Chchd2*^*–/–*^ and *Chchd2*^*+/+*^ MEFs treated with 250 nM rotenone for 72 h and 150 nM actinonin for 118 h. Data are represented as means ± SEM; *n* = 4 per ge*n*otype; **p < 0.01.

To investigate whether the upregulation of the CHCHD2-CHCHD10 complex leads to a selective advantage in damaged mitochondria, we assessed the growth and survival rate of primary MEFs under different stress conditions. We hence generated and analyzed four *Chchd2* knockout and four control MEF lines (Fig. 7A). The loss of CHCHD2 caused ~50% decrease in the mtDNA-encoded OXPHOS subunit MT-COI in primary MEFs (Fig. 7B), without affecting the enzymatic activities of complexes IV or I (Fig. S8A). Notably, there was a significant reduction (~50%) in cell proliferation in galactose medium, where cells were forced to rely on OXPHOS for ATP production (Fig. 7C). We measured how treatment with the complex I inhibitor rotenone or the antibiotic actinonin, which compromises mitochondrial translation, impacts CHCHD2 and CHCHD10 levels. Western blot analyses showed that CHCHD2 and CHCHD10 were upregulated 24 h after rotenone treatment (Fig. S8B), whereas exposure to actinonin caused CHCHD2 and CHCHD10 accumulation only after 72 h (Fig. S8C). Based on these observations, we analyzed *Chchd2* knockout and control MEFs exposed to rotenone for 24 to 72 h and actinonin for 72 to 118 h. We found that CHCHD2 ablation did not prevent CHCHD10 accumulation in response to mitochondrial insults, but rather enhanced it (Fig. 7D, E), suggesting that the upregulation of these proteins is beneficial to counteract the mitochondrial impairment. To test this hypothesis, we measured cell viability after rotenone and actinonin treatments and found that both agents caused a substantial decrease in cell viability in control MEFs (Fig. 7F, G). Notably, cells lacking CHCHD2 were significantly more sensitive to both treatments (Fig. 7F, G).

## Discussion

Several mutations in *CHCHD2* and *CHCHD10* have been found in patients with neurodegenerative diseases. However, it remains unclear whether the pathophysiology is driven by gain-of-function or loss-of-function mechanisms. Research efforts have largely focused on understanding the impact of mutations causing the accumulation of toxic species [53–55], while the physiological function of CHCHD2 and CHCHD10 remains poorly understood. This study explores the role of CHCHD2 using mammalian systems and examines the consequences of its loss on mouse gross and molecular phenotypes. We here show that adult male mice lacking CHCHD2 manifest a modest decrease in muscle strength and gait abnormalities, likely caused by the decreased DA levels in the striatum. While our results diverge from previous reports and mouse models [24, 25], these discrepancies may lie in the methodological differences, such as targeting strategies, genetic backgrounds, mouse age, cohort sizes, and sex stratification. Notably, our use of larger cohorts may have enhanced our ability to detect subtle, sex-specific phenotypes. Despite these contrasts, earlier studies are consistent with our findings in suggesting that CHCHD2 deficiency does not impact the early development of the nigrostriatal system. In line with this hypothesis, single-cell RNAseq data have shown that mouse ventral midbrain neurons express *CHCHD2* and *CHCHD10* at low levels during embryonic and perinatal stages, while both genes are strongly upregulated in adulthood [56]. This temporal expression pattern may explain why *CHCHD2* loss does not affect juvenile mice but leads to defects in adults, offering a plausible explanation for the onset of neurodegenerative disorders in humans.

Although CHCHD2 was initially identified as an OXPHOS regulator [57], there is still no clear consensus on whether CHCHD2 loss affects respiratory capacity. Previous studies suggest CHCHD2 modulates complex IV activity by acting as a transcription factor under stress conditions [58], although its nuclear localization remains debated. Other reports indicate that CHCHD2 disruption mildly reduces ATP levels in fly muscle [23], impairs Complex I assembly in zebrafish [59], and decreases oxygen consumption in glioblastoma cells [60]. However, CHCHD2-deficient HEK293T cells maintain normal respiration [50]. Our data demonstrate that the loss of CHCHD2 does not impair mitochondrial respiration in differentiated mouse tissues or primary MEFs, suggesting that CHCHD2 is dispensable for tissue bioenergetics under physiological conditions.

Mutations in *CHCHD2* and *CHCHD10* have been predominantly associated with cristae abnormalities [20, 61]. However, the hearts of one-year-old CHCHD2-knockout mice do not exhibit any disruptions in cristae organization but present a clear accumulation of lipid vacuoles. Additionally, the brains of these animals showed altered lipid content, with significant changes observed in myelin-associated sphingolipid species within the cortex and dorsal striatum, whereas few phospholipid species were dysregulated in these regions. In line with our findings, a recent study reported the presence of phospholipid alterations in the heart of CHCHD10 mutant mice [62]. It is also noteworthy that another member of the CHCH-domain protein family, Mdm35 (TRIAP in humans), is involved in the regulation of lipid content by mediating the transfer of phospholipids between the outer and inner mitochondrial membranes [63, 64]. Although the underlying molecular mechanisms remain unknown, these data suggest that CHCHD2 is involved in maintaining lipid homeostasis.

In proliferative cells, CHCHD2 and CHCHD10 are mainly present as monomers or dimers [13, 14]. A similar distribution has been observed for CHCHD10 in zebrafish larvae [59], raising questions about the functional relevance of the CHCHD2-CHCHD10 complex. We here provide compelling evidence that in differentiated tissues CHCHD2 and CHCHD10 exist exclusively as a complex of ~100–140 kDa, suggesting that these proteins do not function as free monomers. Contrary to earlier reports [50, 65], we demonstrate that CHCHD10 oligomerizes without CHCHD2, which may explain how these functionally redundant proteins compensate for one another. The analysis of CHCHD2 and CHCHD10 interactomes confirmed significant interactions with the mitochondrial proteolytic system, including the SPY complex, PHB complex, and GHITM (TMBIM5 or MICS1). These interactions may account for rapid turnover of CHCHD2 and CHCHD10, as their half-lives in cell lines range from 2 to 5 h [14, 66]. This implies that CHCHD2 and CHCHD10 are constantly degraded, and their levels are finely tuned under physiological conditions. In response to mitochondrial stress, CHCHD2 and CHCHD10 accumulate, likely increasing protein stability [23, 50]. Our data reveal that this process is highly conserved in vivo and represents a common response across various tissues and organs affected by mitochondrial dysfunction. Strikingly, this increase correlates with the severity of mitochondrial defects, leading to the formation of larger and more abundant protein complexes. In MEFs, the absence of CHCHD2 does not prevent CHCHD10 accumulation under mitochondrial stress but worsens cell survival. This suggests that CHCHD2 upregulation serves as a protective mechanism under pathological conditions, in line with two recent reports showing that CHCHD2 confers protection to Huntington’s disease models [67, 68].

Overall, the present study advances our understanding of how loss-of-function mutations in *CHCHD2* contribute to disease pathology, although the mechanisms regulating CHCHD2 levels and assembly remain to be elucidated.

## Supplementary information

Supplementary figures

Reproducibility checklist

Uncropped western blots

## Data Availability

All data needed to evaluate the conclusions in the paper are present in the paper and/or the Supplementary Materials.
