# Supplementary material for: The CHCHD2-CHCHD10 protein complex is modulated by mitochondrial dysfunction and alters lipid homeostasis in the mouse brain
Source: Cell Death Dis. 2025 Oct 6;16(1):693. doi: 10.1038/s41419-025-08030-z (PMC12501252; doi:10.1038/s41419-025-08030-z)
Supplement: Supplementary file 1 — Supplementary figures [file 41419_2025_8030_MOESM1_ESM.pdf]

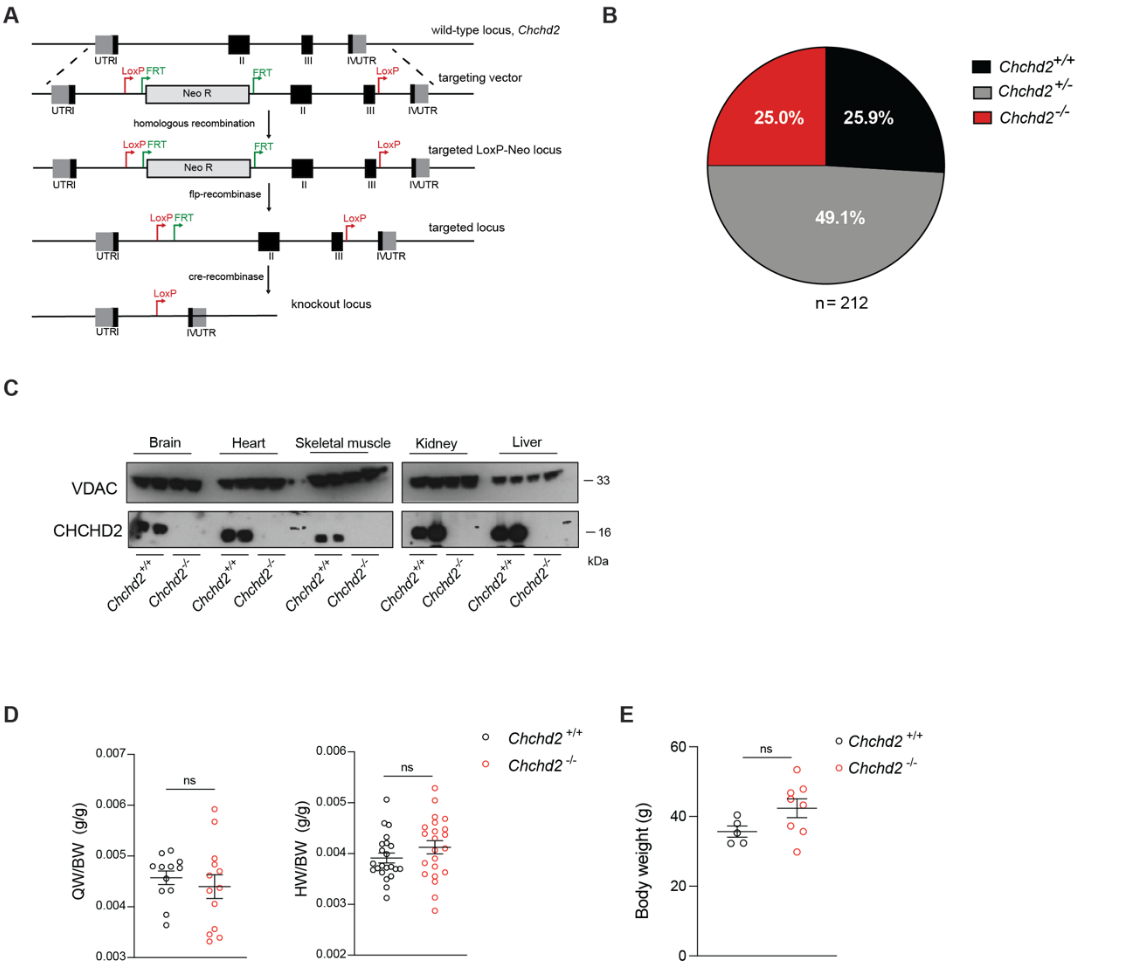

Fig. S1

Fig. S1. Generation of *Chchd2* Knockout mice. A. Targeting strategy for disruption of the *Chchd2* gene. LoxP sites flanking exons 2 and 3 of the *Chchd2* gene together with neomycin selection marker (Neo R) were inserted into the mouse genome by homologous recombination. The Neo R cassette was excised by mating with flp-recombinase mice to obtain heterozygous floxed *Chchd2* mice (*Chchd2* $^{+/loxP}$). Whole-body *Chchd2* knockout mice were obtained by crossing *Chchd2* $^{+/loxP}$ with Beta-actin cre-recombinase mice. **B.** Mendelian distribution of offspring obtained by crossing heterozygous knockout *Chchd2* (*Chchd2*$^{+/-}$) males and females. n = 212; *Chchd2*$^{-/-}$ n = 53; *Chchd2*$^{+/-}$ n = 104; *Chchd2*$^{+/+}$ n =55. **C.** Western blot of the CHCHD2 protein levels in mitochondrial extracts from different tissues of one-month-old *Chchd2*$^{+/+}$ and *Chchd* $^{-/-}$ mice. VDAC was used as loading control. **D.** Quadriceps (QW) and heart (HW) weight to BW (g/g) ratios in males at 12 months of age. Data are represented as means ± SEM; n>18, ns not significant. **E.** BW (g) of female *Chchd2*$^{-/-}$ and *Chchd2*$^{+/+}$ mice at the age of 20 months. Data are represented as means ± SEM; n≥ 5, ns not significant.

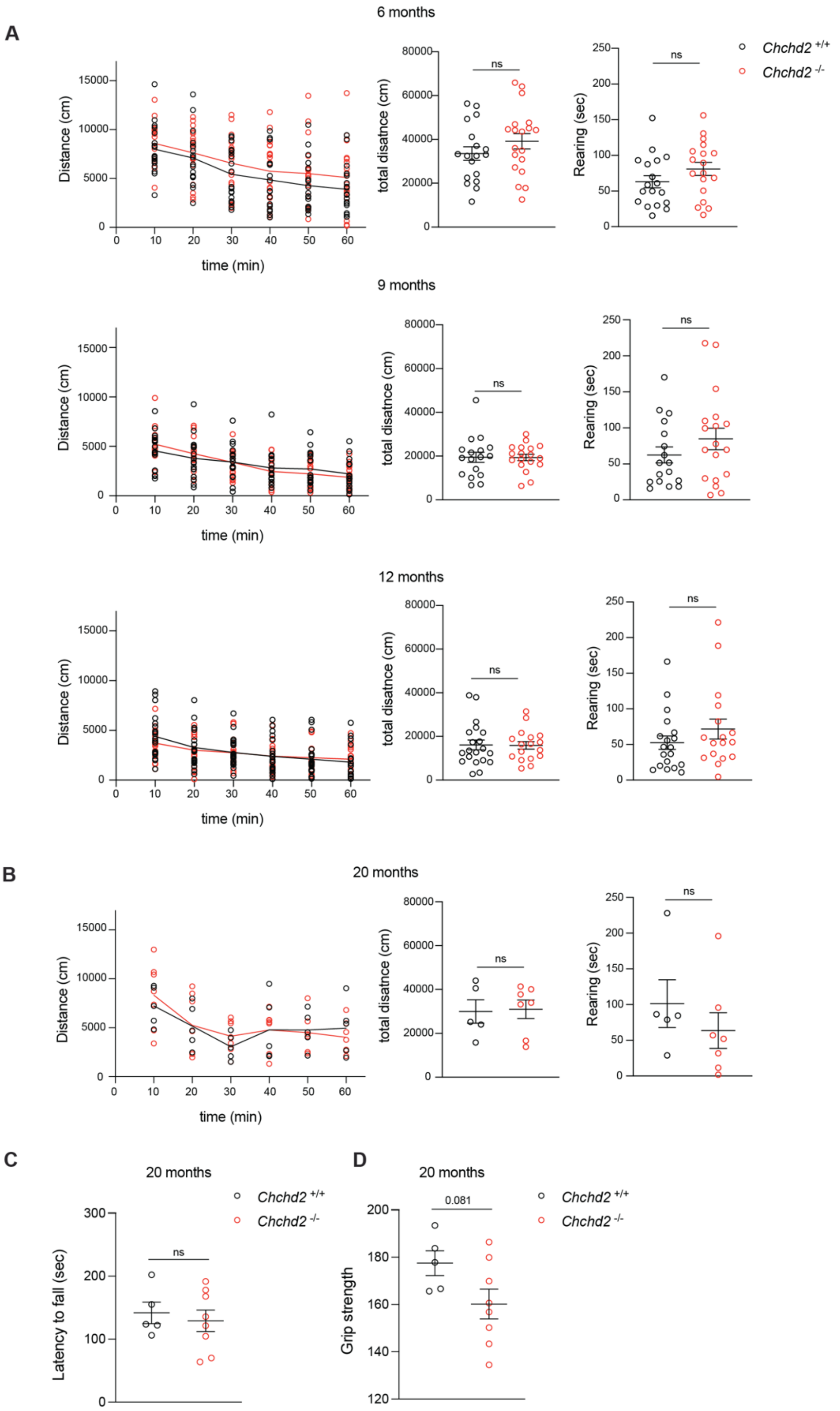
A
6 months
Distance (cm)
time (min)
total disatnce (cm)
Rearing (sec)
ns
Chchd2 +/+
Chchd2 -/-
9 months
12 months
B
20 months
C
20 months
Latency to fall (sec)
D
20 months
Grip strength
0.081

Fig. S2

Fig. S2. Spontaneous locomotion is not affected by the loss of CHCHD2 in both male and female mice. Spontaneous locomotor activity measured as distance and rearing, in open field arena over 60 minutes in **A.** *Chchd2*$^{-/-}$and *Chchd2*$^{+/+}$ male mice at the age of 6, 9 and 12 months. Data are represented as means ± SEM; n ≥ 16, ns not significant. **B.** in *Chchd2*$^{-/-}$ and *Chchd2*$^{+/+}$ female mice at the age of 20 months. Data are represented as means ± SEM; *n* = 5–7; ns not significant. **C.** Motor coordination measured as latency to fall (in s) from the rod and **D.** Muscular strength (in g) measured by grip strength test in 20-month-old female mice. Data are represented as means ± SEM *n* = 5–7; ns not significant.

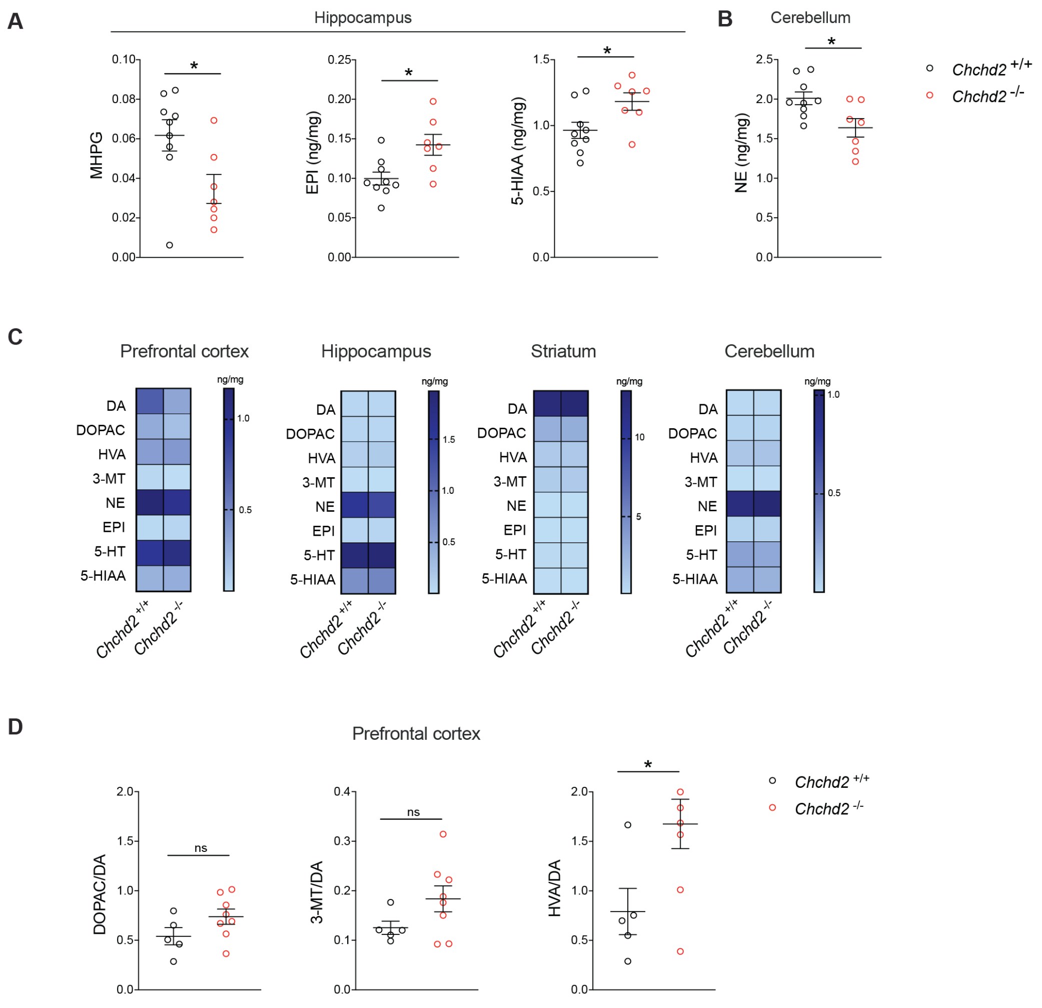

Fig. S3

Fig. S3. Analysis of monoamine neurotransmitters in the brain of male and female mice lacking CHCHD2. A. Hippocampal levels (ng/mg) of 3-Methoxy-4-hydroxyphenylglycol (MHPG), epinephrine (EPI) and 5-Hydroxyindoleacetic acid (5-HIAA) **B.** Cerebellar levels (ng/mg) of NE in *Chchd2*$^{-/-}$ and *Chchd2*$^{+/+}$ male mice at the age of 12 months. Data are represented as means± SEM; n ≥ 7, *p < 0.05, ns not significant. **C.** Heatmaps showing the levels (ng/mg) of DA, NE, 5-HT and their metabolites in the prefrontal cortex, hippocampus, striatum and cerebellum of *Chchd2*$^{-/-}$and *Chchd2*$^{+/+}$ female mice at the age of 20 months. **D.** DA turnover ratios (DOPAC/DA, 3-MT/DA and HVA/DA) in the prefrontal cortex of *Chchd2*$^{-/-}$and *Chchd2*$^{+/+}$ female mice at the age of 20 months. Data are represented as means ± SEM *n* = 5–7; *p < 0.05, ns not significant.

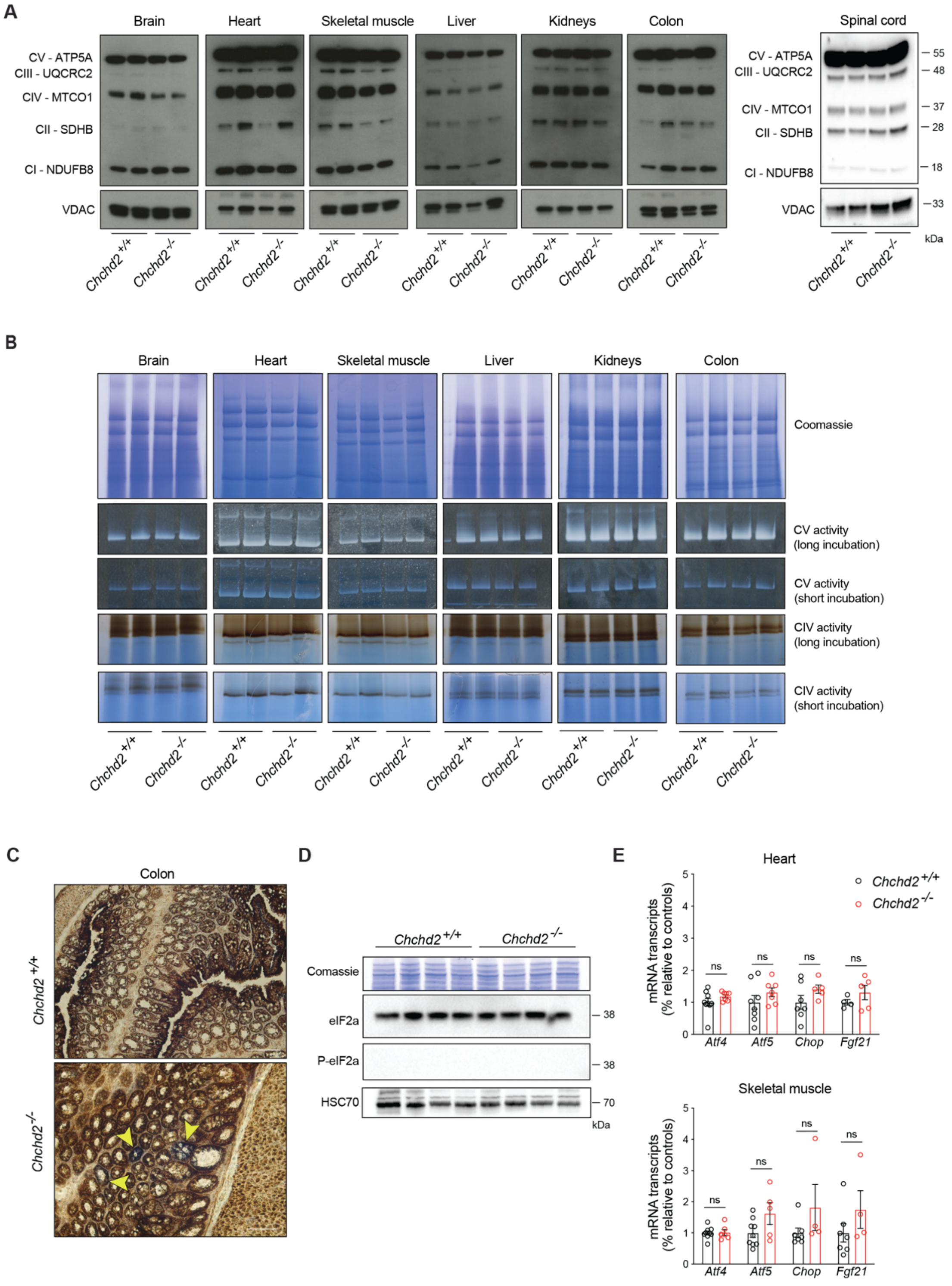
A
Brain
Heart
Skeletal muscle
Liver
Kidneys
Colon
Spinal cord
CV - ATP5A
CIII - UQCRC2
CIV - MTCO1
CII - SDHB
CI - NDUFB8
VDAC
55
48
37
28
18
33
kDa
Chchd2 +/+
Chchd2 -/-
B
Coomassie
CV activity (long incubation)
CV activity (short incubation)
CIV activity (long incubation)
CIV activity (short incubation)
C
Colon
D
Comassie
eIF2a
P-eIF2a
HSC70
38
70
E
Heart
Skeletal muscle
mRNA transcripts (% relative to controls)
ns
Atf4
Atf5
Chop
Fgf21
0
1
2
3
4
5

Fig. S4

Fig. S4. CHCHD2 loss does not compromise OXPHOS nor promote integrated stress response. Western blot analysis of steady-state levels of **A.** OXPHOS subunits in mitochondria isolated from different tissues of *Chchd2*$^{-/-}$ and *Chchd2*$^{+/+}$ male mice at the age of 12 months. VDAC was used as loading control. **B.** Complex IV and V in-gel activities performed on mitochondria isolated from different tissues of *Chchd2*$^{-/-}$ and *Chchd2*$^{+/+}$ male mice at the age of 12 months. BN-PAGE stained with Coomassie or incubated with substrates for detecting the in-gel activity of the indicated OXPHOS complexes. **C.** Cytochrome c oxidase and succinate dehydrogenase (COX/SDH) double-labelling enzyme histochemistry of colon in *Chchd2*$^{-/-}$ and *Chchd2*$^{+/+}$ mice at the age of 12 months (Scale bar: 100 μm). COX-deficient colonic crypts (in blue) are indicated by the yellow arrows. **D.** Western blot analysis of eIF2α phosphorylation at serine 51, a key integrated stress response event triggered by the cytosolic DELE1 accumulation, and total eIF2α levels in heart tissue from *Chchd2*$^{-/-}$ and *Chchd2*$^{+/+}$ male mice at the age of 12 months. HSC70 and Coomassie staining were used as loading controls. *n*=4 per genotype. **E.** *Atf4, Atf5, Chop* and *Fgf21* transcript levels measured by qPCR on RNA isolated from heart and skeletal muscle (quadriceps) in mice at 12 months of age. Data are represented as means ± SEM; *n*=5; ns not significant.

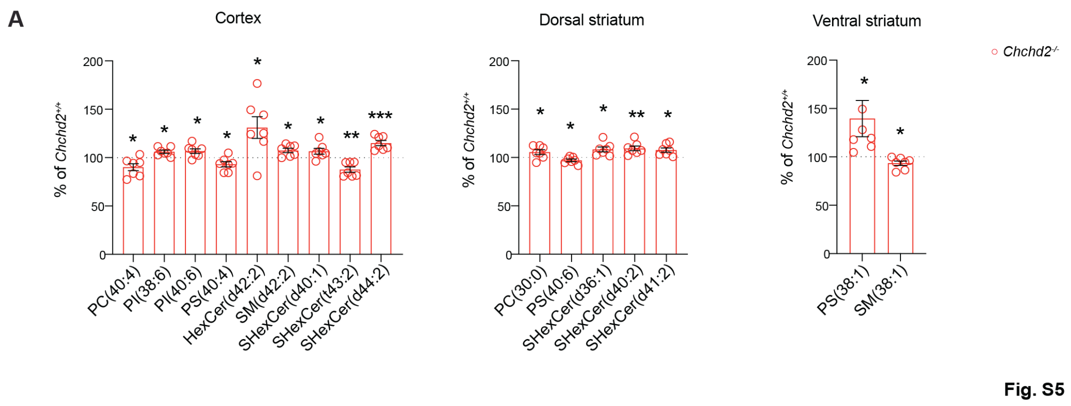

Fig. S5. Mild alterations in lipid homeostasis in male mice lacking CHCHD2. A. Relative quantification of affected lipids in different brain regions of *Chchd2*-/- male mice at the age of 12 months. Data are represented as means ± SEN; *n* ≥ 7; *p< 0.05, **P < 0.01.

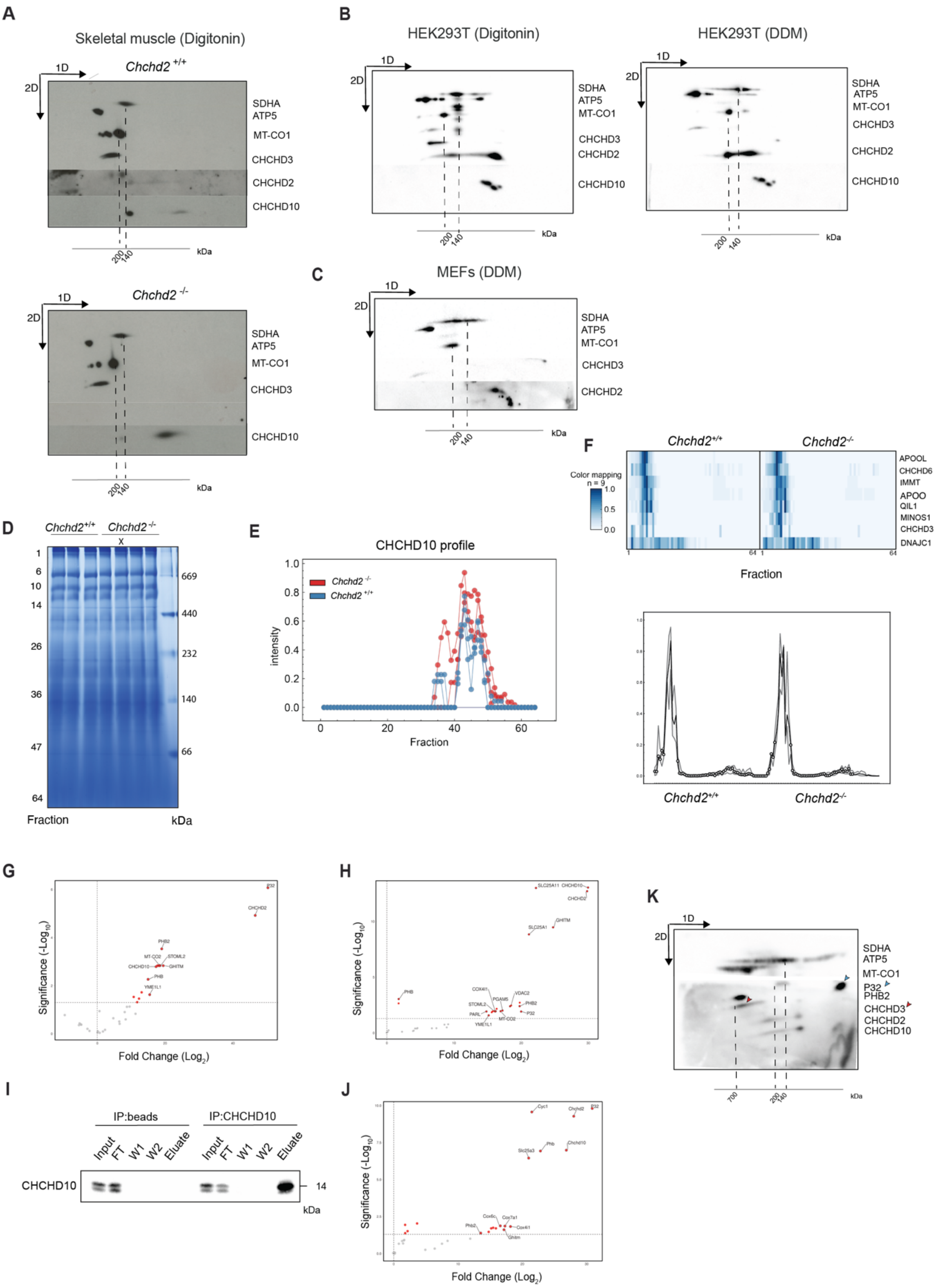
A
Skeletal muscle (Digitonin)
Chchd2 +/+
Chchd2 -/-
1D
2D
SDHA
ATP5
MT-CO1
CHCHD3
CHCHD2
CHCHD10
200
140
kDa
B
HEK293T (Digitonin)
HEK293T (DDM)
C
MEFs (DDM)
D
Chchd2+/+
Chchd2-/-
1
6
10
14
26
36
47
64
669
440
232
140
66
Fraction
kDa
E
CHCHD10 profile
Chchd2 -/-
Chchd2 +/+
intensity
Fraction
F
Color mapping
n = 9
APOOL
CHCHD6
IMMT
APOO
QIL1
MINOS1
CHCHD3
DNAJC1
Fraction
G
H
Significance (-Log10)
Fold Change (Log2)
I
IP:beads
IP:CHCHD10
Input
FT
W1
W2
Eluate
CHCHD10
14
kDa
J
K
SDHA
ATP5
MT-CO1
P32
PHB2
CHCHD3
CHCHD2
CHCHD10
700
200
140
kDa

Fig. S6

Fig. S6. CHCHD2 and CHCHD10 are not MICOS subunits. 2D-PAGE of mitochondria isolated **A.** from skeletal muscle of *Chchd2*$^{-/-}$ and *Chchd2*$^{+/+}$ male mice at the age of 12 months. Mitochondria were solubilized using 1% (w/v) digitonin. **B.** from HEK293T cells and solubilized using either with 1% (w/v) DDM or digitonin. **C.** from MEFs and solubilized using 1% (w/v) DDM. The position of SDHA, ATP5, MT-COI, and CHCHD3 corresponds to the size of their different protein complexes (in kDa). **D.** Mitochondria isolated from skeletal muscle of *Chchd2*$^{-/-}$ and *Chchd2*$^{+/+}$ male mice at the age of 12 months (n=3-4) were solubilized using 1% (w/v) digitonin and resolved on self-made 3%– 13% gels. **E.** Migration profiles of CHCHD10 obtained from complexome profiling analysis of mitochondria isolated from skeletal muscle of *Chchd2*$^{-/-}$ mice at the age of 12 months. n=3. **F.** Heatmaps (upper panel) and migration profiles (lower panel) of the MICOS complex subunits obtained by complexome profiling performed on mitochondria isolated from skeletal muscle of *Chchd2*$^{-/-}$ and *Chchd2*$^{+/+}$ male mice at the age of 12 months. n=3. **G–H.** Volcano plots showing the proteins identified by mass spectrometry in **G.** CHCHD2 and **H**. CHCHD10 pull-down experiments from HEK293T mitochondrial extracts. **I.** Representative Western blot analysis of CHCHD10 immunoprecipitation experiments performed on mitochondria isolated from the heart (n = 4 IP and control samples from 4 wild-type mice). CHCHD10 detection in the antibody-bound (ab), flow-through (FT), and wash (W) fractions. **J.** Volcano plot showing proteins identified by mass spectrometry in CHCHD10 pull-down experiments performed on heart mitochondria**. K.** Two-dimensional PAGE analysis of mitochondria isolated from the hearts of 12-month-old *Chchd2*$^{+/+}$ male mice. Mitochondria were solubilized with 1% (w/v) DDM. The migration positions of SDHA, ATP5, MT-CO1, and CHCHD3 (red arrows) correspond to their respective protein complexes (in kDa). P32 is marked with blue arrows.

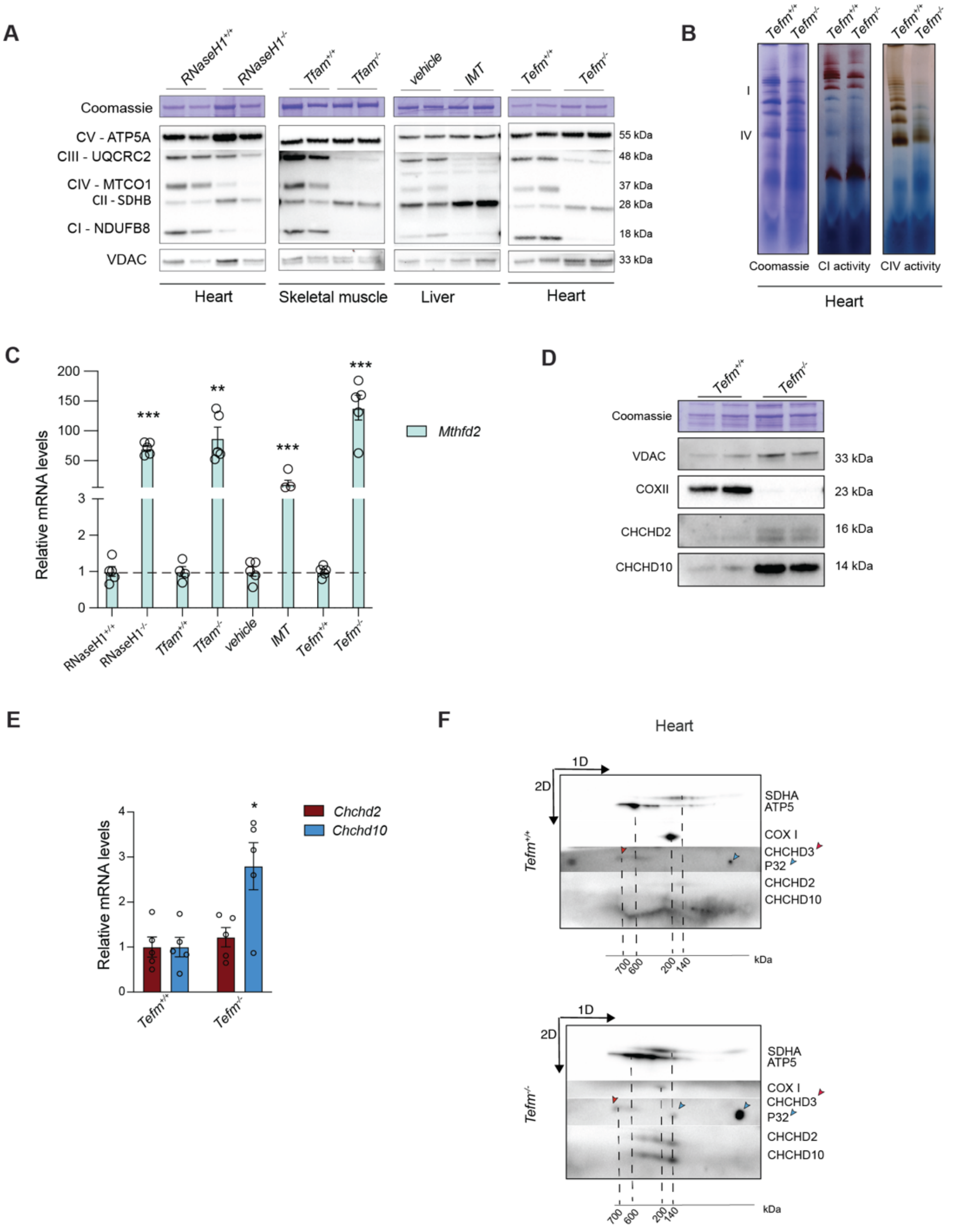

Fig. S7

Fig. S7. CHCHD2 and CHCHD10 form a larger and more abundant complex in response to mitochondrial dysfunction. A. Western blot analysis of OXPHOS subunits levels in mitochondria isolated from mouse models with impaired mitochondria. VDAC and Coomassie staining were used as loading controls. **B.** Complex IV and V in-gel activities performed on mitochondria isolated from heart of *Tefm* knockout mice at 8 weeks of age. **C.** *Mthfd2* mRNA levels measured by qPCR from RNA isolated from mouse tissues with mitochondrial dysfunction. Data are represented as mean ± SEM. n= 5 mice for each genotype, *p < 0.05, **p < 0.01, ***p< 0.001. **D.** Western Blot analysis of CHCHCD2 and CHCHD10 steady-state levels in mitochondria isolated from heart of *Tefm* knockout mice at 8 weeks of age. **E.** *Chchd2* and *Chchd10* mRNA levels measured by qPCR from RNA isolated from heart of *Tefm* knockout mice at 8 weeks of age. Data are represented as mean ± SEM. n= 5 mice for each genotype, *p < 0.05. **F.** 2D-PAGE of mitochondria isolated from skeletal muscle of from heart of *Tefm* knockout mice at 8 weeks of age. Mitochondria were solubilized using 1% (w/v) DDM. The position of SDHA, ATP5, MT-CO1, and CHCHD3 (indicated by a red arrow) corresponds to the size of their different protein complexes (in kDa). P32 is indicated by blue arrows.

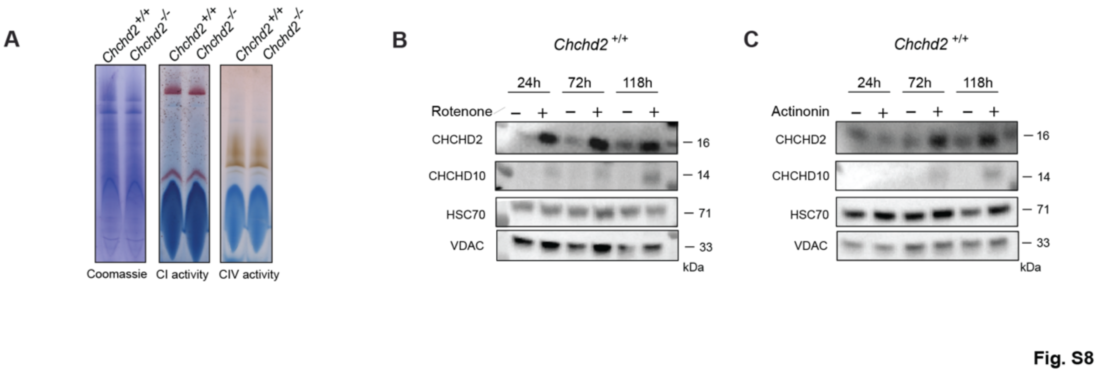

Fig. S8. CHCHD2 and CHCHD10 accumulate in MEFs treated with rotenone or actinonin. A. Complex I and IV in-gel activities performed on mitochondria isolated from *Chchd2*$^{-/-}$ and *Chchd2*$^{+/+}$ MEFs. BN-PAGE stained with Coomassie or incubated with substrates for detecting the activity of the indicated OXPHOS complexes. **B-C.** Representative Western Blot analysis of CHCHCD2 and CHCHD10 steady-state levels in total extract from primary *Chchd2*$^{+/+}$ MEFs treated with 250 nM rotenone for 24, 48 and 72 hours and 150 nM actinonin for 24, 72 and 118 hours. HSC70 and Coomassie were used as loading controls.

Tables:

Table S1: Primary antibody list

Antigen	Source	Company	Catalog nr	Primary dilution	Molecular weight (kDa)
**MitoProfile Human**	Mouse	Abcam	ab110411	(1:3000)	55-48-37-28-18
**CHCHD2**	Rat	Helmholtz Munich	CCHD 16D9	Supernatant	16
**CHCHD2**	Rabbit	ProteinTech	19422-I-AP	(1:500)	16
**CHCHD10**	Rabbit	Sigma	HPA003440	(1:3000)	14
**CHCHD10**	Rabbit	ProteinTech	19424-I-AP	(1:3000)	15
**CHCHD3**	Rabbit	ProteinTech	25625-1-AP	(1:3000)	26
**VDAC**	Mouse	Abcam	MSA03/ab14734	(1:5000)	33
**Mitofilin (Mic60)**	Rabbit	ProteinTech	10179-1-AP	(1:3000)	80-90
**GHITM (MICS1)**	Rabbit	ProteinTech	16296-1-AP	(1:3000)	38
**HSP60**	Mouse	Enzo Lifesciences	AB1-SPA-807-E	(1:2000)	60
**HSC70**	Mouse	Santa Cruz	sc-7298	(1:3000)	70
**OPA1**	Rabbit	Abcam	ab42364	(1:2000)	90-100
**C1QBP (p32)**	Rabbit	Sigma	HPA026483	(1:2000)	32
**YME1L1**	Rabbit	ProteinTech	11510-1-AP	(1:1000)	63
**PHB2**	Rabbit	Abcam	ab75766	(1:1000)	30
**STOML2**	Rabbit	ProteinTech	10348-1-AP	(1:1000)	39
**MT-CO2**	Rabbit		Antisera	(1:5000)	23
**ATP5A**	Mouse	Abcam	ab147448	(1:5000)	55
**SDHA**	Mouse	Abcam	ab14715	(1:5000)	70
**MT-CO1**	Mouse	Invitrogen	459600	(1:1000)	37
**eIF2α**	Rabbit	Cell Signaling	5324T	(1:2000)	38
**Phospho-eIF2α (Ser51)**	Rabbit	Cell Signaling	3398T	(1:1000)	38
**TH**	Mouse	Chemicon	MAB318	(1:500-1000)	

Table S2. List of TaqMan probes

GENE	*TAQMAN PROBE ID*
Chchd2	Mm01621550_s1
Chchd10	Mm07302070_g1
Mthfd2	Mm00485276_m1
B- Actin	Mm01205647_g1
Mt-Co1	Mm04225243_g1
Cytb	Mm04225271_g1
Nd1	Mm04225274_s1
Nd4	Mm04225294_s1
Atf4	Mm00515325_g1
Atf5	Mm04179654_m1
Chop Or Ddit3	Mm01135937_g1
Fgf21	Mm00840165_g1
18s rRNA	Mm03928990_g1

Table S3. Identified interactors of endogenous CHCHD2 in HEK293T cells

Gene name	***-Log(P-value)***	***lof2FC***	***Protein names***
C1QBP	6,075158655	50,33133443	Complement component 1 Q subcomponent-binding protein, mitochondrial
CHCHD2	4,924605406	46,57842827	Coiled-coil-helix-coiled-coil-helix domain-containing protein 2, mitochondrial
WARS2	11,28814824	21,0234251	Tryptophan--tRNA ligase, mitochondrial
VDAC1	13,49487394	20,67803733	Voltage-dependent anion-selective channel protein 1
POLDIP2	12,47333063	19,60814031	Polymerase delta-interacting protein 2
GHITM	2,834959277	19,44199308	Growth hormone-inducible transmembrane protein
PHB2	3,531659873	19,00003929	Prohibitin-2
STOML2	2,836382307	18,47478898	Stomatin-like protein 2, mitochondrial
MT-CO2	2,839913569	18,36949666	Cytochrome c oxidase subunit 2
SLC25A5	2,830006645	18,11589368	ADP/ATP translocase 2;ADP/ATP translocase 2, N-terminally processed
SLC25A3	2,833964049	17,96081797	Phosphate carrier protein, mitochondrial
SFXN1	2,837556211	17,75948079	Sideroflexin-1
CHCHD10	2,791752512	17,32434845	Coiled-coil-helix-coiled-coil-helix domain-containing protein 10, mitochondrial
PHB	2,264291177	14,97139657	Prohibitin

Table S4. Identified interactors of endogenous CHCHD10 in HEK293T cells

Gene names	*-logP*	*logFC*	*Protein names*
CHCHD10	13,0391499	29,95812511	Coiled-coil-helix-coiled-coil-helix domain-containing protein 10, mitochondrial
SLC25A11	13,00038678	22,20192003	Mitochondrial 2-oxoglutarate/malate carrier protein
CHCHD2	12,70984575	29,81633472	Coiled-coil-helix-coiled-coil-helix domain-containing protein 2, mitochondrial
GHITM	9,470368995	24,75180149	Growth hormone-inducible transmembrane protein
SLC25A1	8,83733878	21,1583395	Tricarboxylate transport protein, mitochondrial
PHB	3,042742019	1,763472271	Prohibitin
SLC25A6; SLC25A4	2,716956338	19,78556242	ADP/ATP translocase 3;ADP/ATP translocase 3, N-terminally processed;ADP/ATP translocase 1
HSPA9	2,653117582	1,753324604	Stress-70 protein, mitochondrial
ATP5A1	2,464498305	18,51404667	ATP synthase subunit alpha, mitochondrial
PHB2	2,40315313	19,76722679	Prohibitin-2
VDAC2	2,391657039	18,34489145	Voltage-dependent anion-selective channel protein 2
HSP90AA1	2,16463086	16,3484334	Heat shock protein HSP 90-alpha
PGAM5	2,008958022	17,22167692	Serine/threonine-protein phosphatase PGAM5, mitochondrial
ATP5C1	1,958327291	15,98890734	ATP synthase subunit gamma, mitochondrial
MT-CO2	1,949919387	16,92953634	Cytochrome c oxidase subunit 2
COX4I1	1,947517998	16,19647694	Cytochrome c oxidase subunit 4 isoform 1, mitochondrial
SFXN1	1,934530549	15,73735237	Sideroflexin-1
STOML2	1,927370711	16,13535681	Stomatin-like protein 2, mitochondrial
PARL	1,925565354	14,82555056	Presenilins-associated rhomboid-like protein, mitochondrial;P-beta
P32	1,921266933	19,99099941	Complement component 1 Q subcomponent-binding protein, mitochondrial
ATP5B	1,853779802	15,60795879	ATP synthase subunit beta, mitochondrial
YME1L1	1,577678431	15,16261683	ATP-dependent zinc metalloprotease YME1L1

Table S5: Common interactors between CHCHD2 and CHCHD10 in HEK293T cells.

Gene name	*CHCHD10: log2FC*	*CHCHD2: log2FC*
CHCHD10	20,4757069	17,3243484
CHCHD2	19,9869816	46,5784283
GHITM	17,2734874	19,4419931
PHB2	13,8306508	19,0000393
STOML2	13,2640279	18,474789
ATP5C1	13,1421083	8,74178092
SFXN1	12,5898819	17,7594808
MT-CO2	10,6908516	18,3694967
ATP5B	9,81515361	12,305958
C1QBP	9,76848689	50,3313344
ATP5A1	9,63199343	8,08011036
YME1L1	9,50194866	15,4506259
MRPL34	8,96722505	4,72670574
CYC1	2,56963337	13,0757624
ATAD3A	2,49557571	-4,73028011
SLC25A5	1,77081964	18,1158937
HSPA9	1,49343104	0,15775312
PHB	1,43103698	14,9713966
MRPS12	0,65431851	0,06124662
MRPL14	-0,00709872	0,64255511

Table S6: Identified interactors of endogenous CHCHD10 in mouse heart'

Gene name	*-Log(P-value)*	*lof2FC*	*Protein names*
P32	9,804529	30,86223	Complement component 1 Q subcomponent-binding protein, mitochondrial
Cyc1	9,568022	21,46795	Cytochrome c1, heme protein, mitochondrial
Chchd2	9,278912	27,99105	Coiled-coil-helix-coiled-coil-helix domain-containing protein 2
Chchd10	6,98593	26,83668	Coiled-coil-helix-coiled-coil-helix domain-containing protein 10
Phb	6,92927	22,81399	Prohibitin
Slc25a3	6,453271	20,98664	Phosphate carrier protein, mitochondrial
Slc25a4	2,035235	3,629871	ADP/ATP translocase 1
Atp5a1	1,941287	1,7692	ATP synthase subunit alpha, mitochondrial
Cox6c	1,872935	16,57426	Cytochrome c oxidase subunit 6C
Cox7a1	1,857505	17,29191	Cytochrome c oxidase subunit 7A1, mitochondrial
Cox4i1	1,828621	18,15653	Cytochrome c oxidase subunit 4 isoform 1, mitochondrial
Acadvl	1,745051	15,46429	Very long-chain specific acyl-CoA dehydrogenase, mitochondrial
Uqcrc1	1,70501	15,88646	Cytochrome b-c1 complex subunit 1, mitochondrial
Idh2	1,703154	15,15483	Isocitrate dehydrogenase [NADP], mitochondrial

Table S7: Common interactors between CHCHD2 and CHCHD10 in HEK293T cells and mouse heart.

Gene name	*Mean_D2_D10_log2FC_HEC*	*Normalized_CHCHD10_heart: log2FC **
CHCHD10	18,90002765	18,34226878
CHCHD2	33,28270495	18,76342622
GHITM	18,35774025	11,96236694
PHB2	16,41534505	9,464505905
P32	30,04991065	15,08065297
ATP5A1	8,856051895	0,920432265
MRPL34	6,846965395	0,827617998
HSPA9	0,82559208	1,836803009
PHB	8,20121679	18,51328082

Table S8. List of assigned lipid species with high mass accuracy obtained from MALDI-FTICR-MSI data.

Lipid species marked with an asterisk (*) were identified using mass accuracy, MS/MS and by comparing the observed distributions of different adducts of the same molecule across the coronal brain tissue sections.

Lipid Species Assignment	Ion Type	Formula	m/z theoretical	m/z observed	ppm error
SHexCer(t43:2)*	$[M-H]^-$	C49H93NO12S	918.634572	918.63573	1,3
SHexCer(d44:2)*	$[M-H]^-$	C50H95NO11S	916.655308	916.65431	-1,1
SHexCer(d40:1)*	$[M-H]^-$	C46H87NO11S	860.592707	860.59429	1,8
SHexCer(d36:1)*	$[M-H]^-$	C42H81NO11S	806.545757	806.54635	0,7
SHexCer(d41:2)*	$[M-H]^-$	C47H89NO11S	874.608357	874.60697	-1,6
PI(40:6)*	$[M-H]^-$	C49H83O13P	909.549853	909.55148	1,8
PI(38:6)*	$[M-H]^-$	C47H79O13P	881.518553	881.52105	2,8
PS(40:4)*	$[M-H]^-$	C46H82NO10P	838.560358	838.55987	-0,6
PS(40:6)*	$[M-H]^-$	C46H78NO10P	834.529058	834.52877	-0,3
PS(40:6)-2ndisotope	$[M-H]^-$	C46H78NO10P	835.532425	835.53096	-1,8
PS(38:1)*	$[M-H]^-$	C44H84NO10P	816.576008	816.57793	2,4
SM(d42:2)*	$[M+H]^+$	C47H93N2O6P	813.684402	813.68583	1,8
HexCer(d42:2)*	$[M+K]^+$	C48H91NO8K	848.637627	848.63962	2,3
HexCer(d42:2)-2ndisotope	$[M+K]^+$	C48H91NO8K	849.641	849.64136	0,4
PC(40:4)*	$[M+H]^+$	C48H88NO8P	838.632032	838.63221	0,2
PC(40:4)-2ndisotope	$[M+H]^+$	C48H88NO8P	839.635405	839.63472	-0,8
PC(30:0)*	$[M+H]^+$	C38H76NO8P	706.538132	706.53861	0,7
SM(d38:1)*	$[M+K]^+$	C43H87N2O6PK	797.593333	797.59516	2,3
