# Supplementary material for: The CHCHD2-CHCHD10 protein complex is modulated by mitochondrial dysfunction and alters lipid homeostasis in the mouse brain
Source: Cell Death Dis. 2025 Oct 6;16(1):693. doi: 10.1038/s41419-025-08030-z (PMC12501252; doi:10.1038/s41419-025-08030-z)

Original WB images Fig. S1C

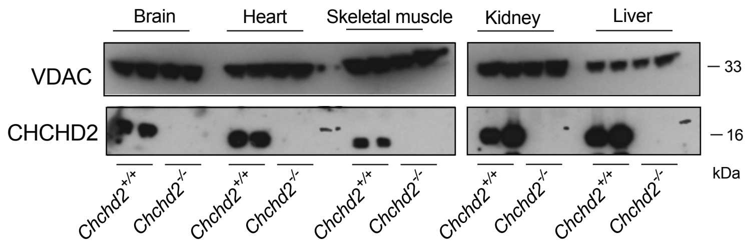

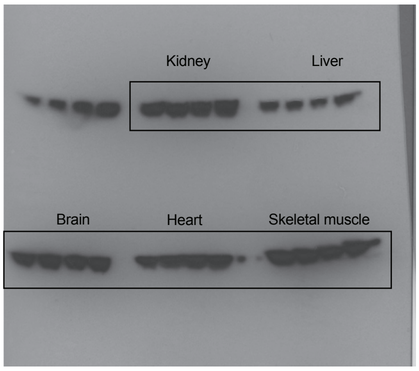

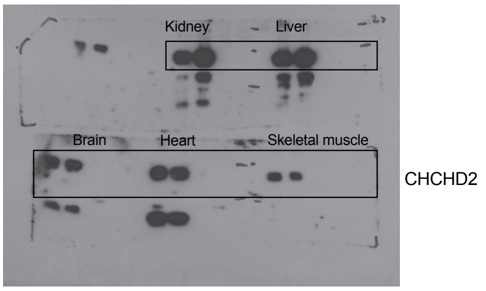

Original WB images Fig. S4A

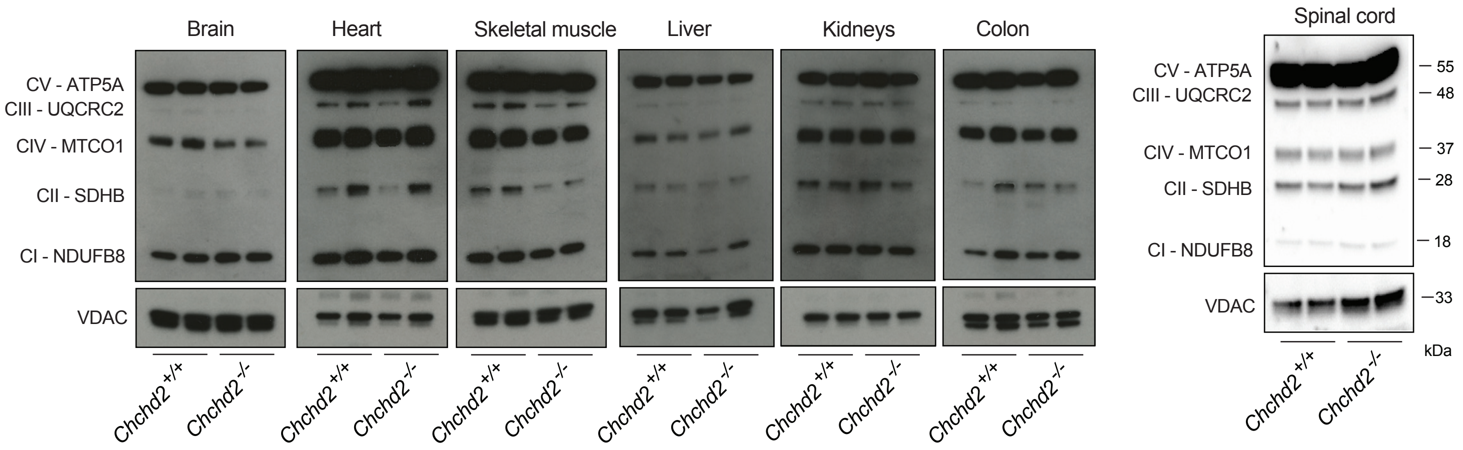

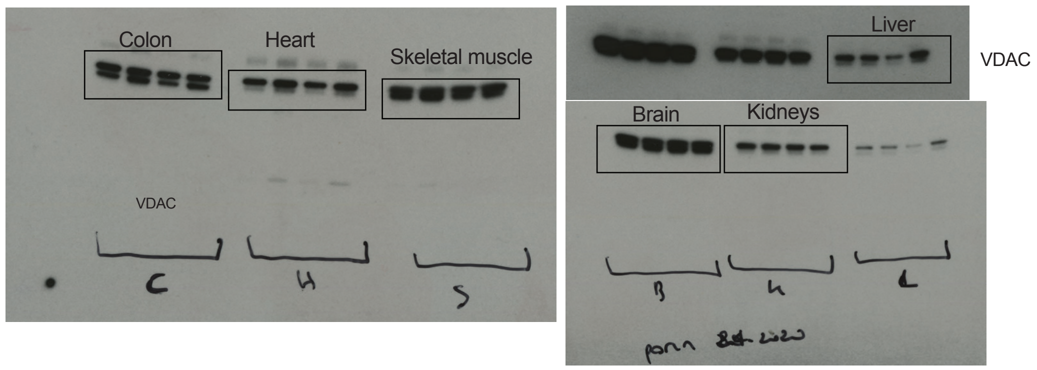

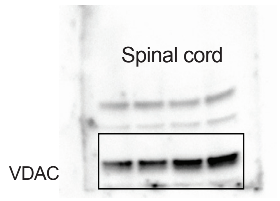

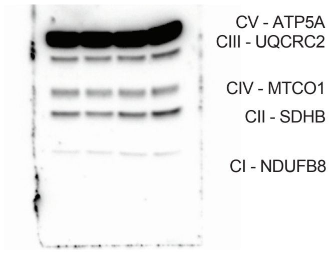

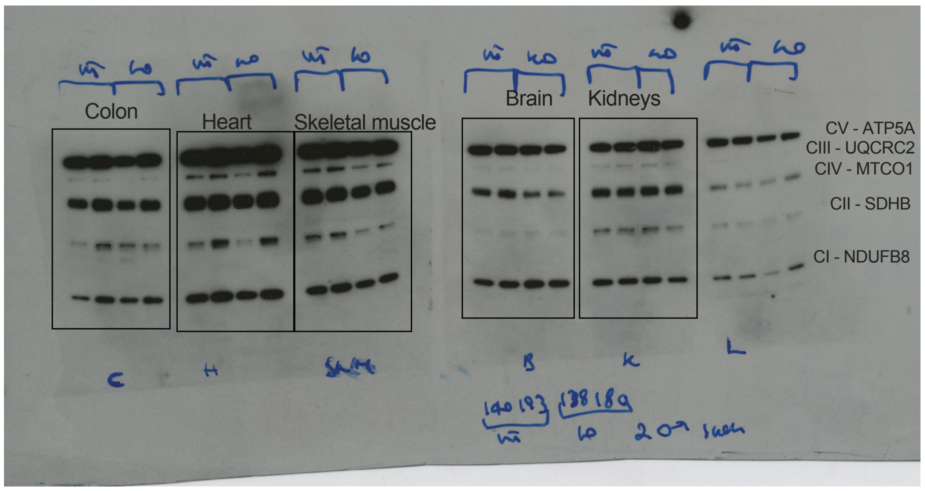

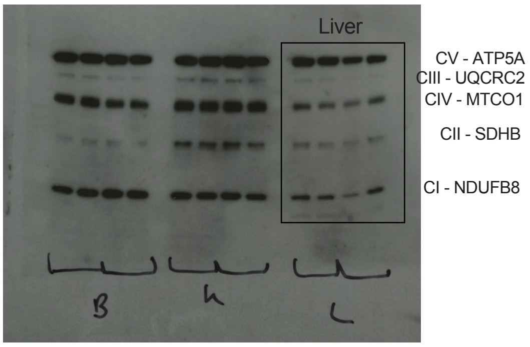

Original WB images Fig. 5C

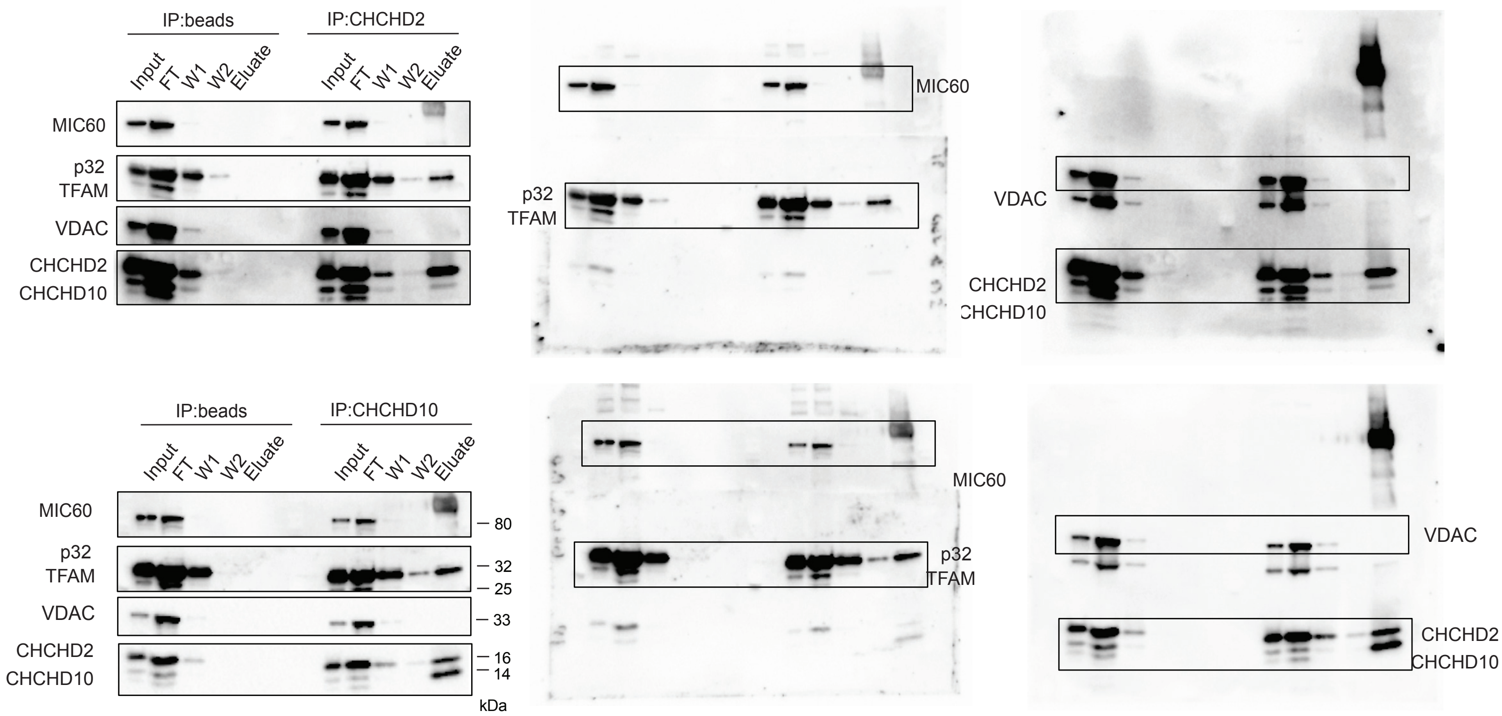

Original WB images Fig.S6A

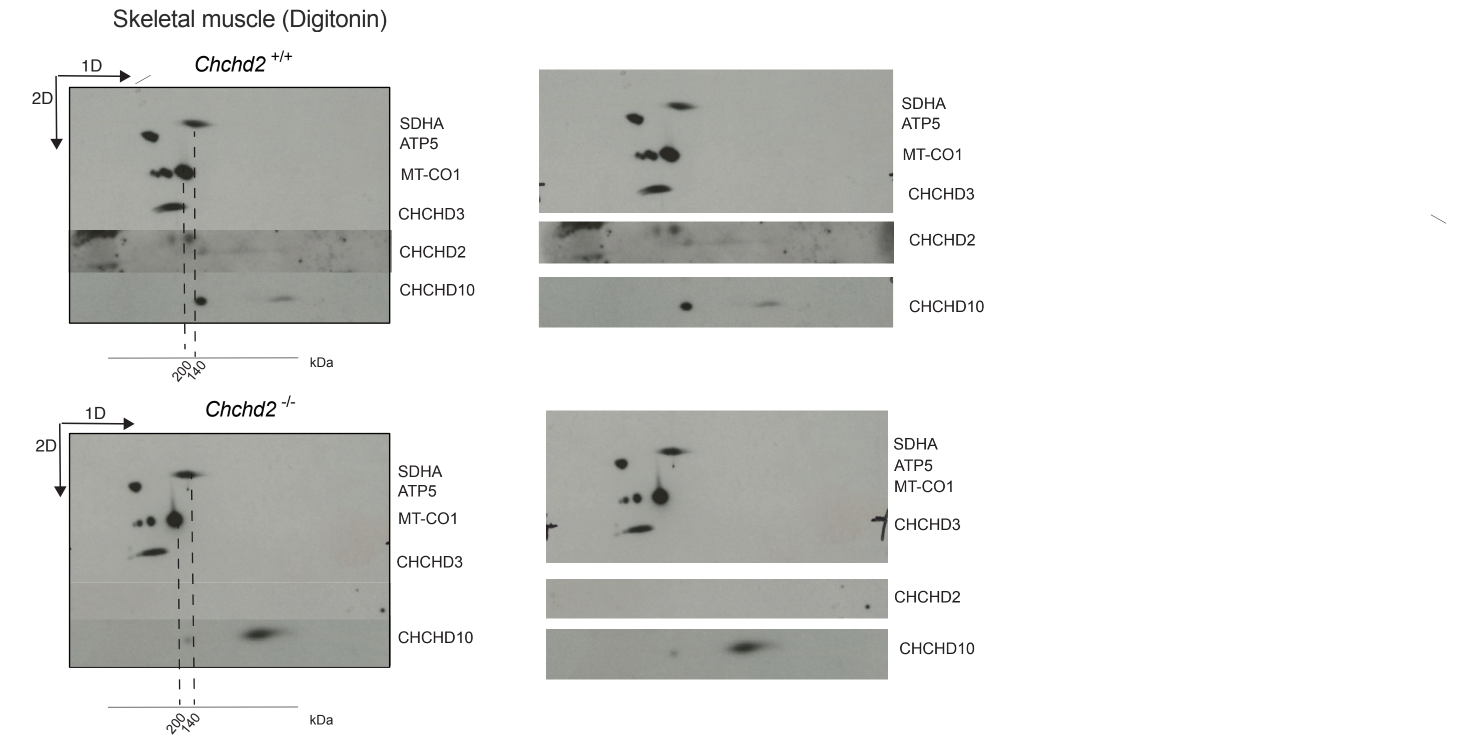

Original WB images Fig.S6B

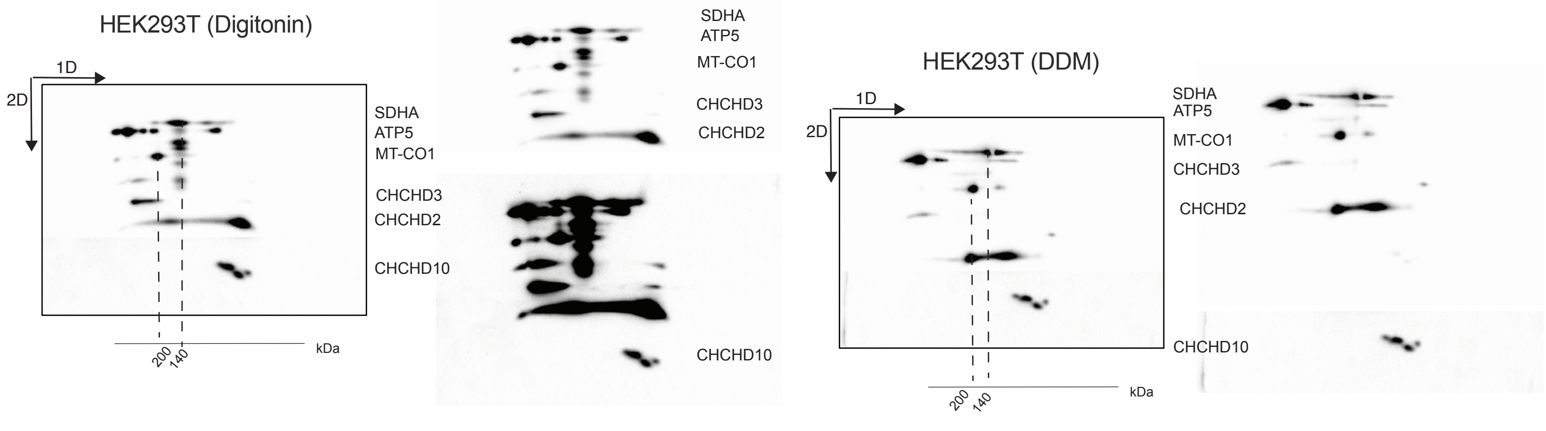

Original WB images Fig.S6C

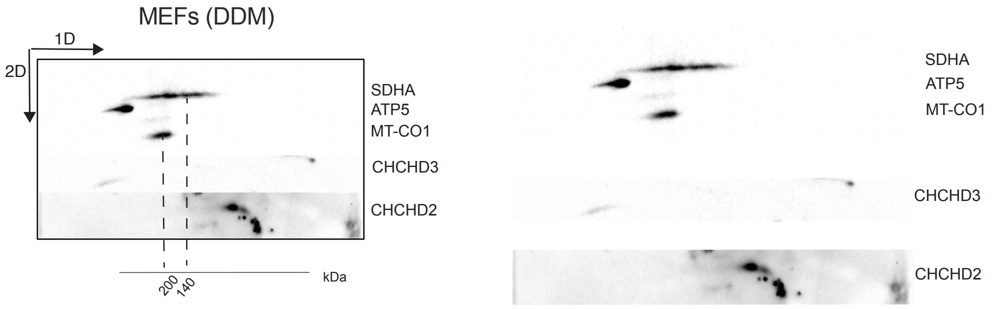

Original WB images Fig.S6I

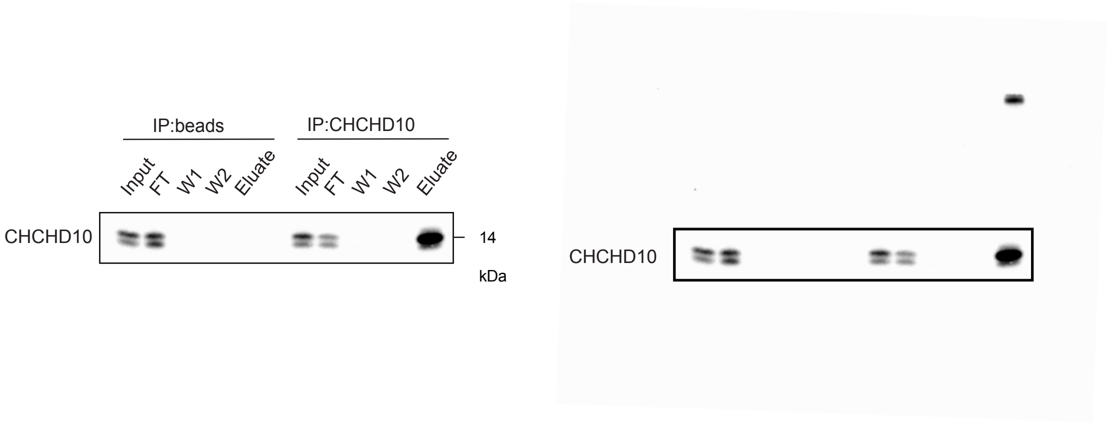

Original WB images Fig.S6K

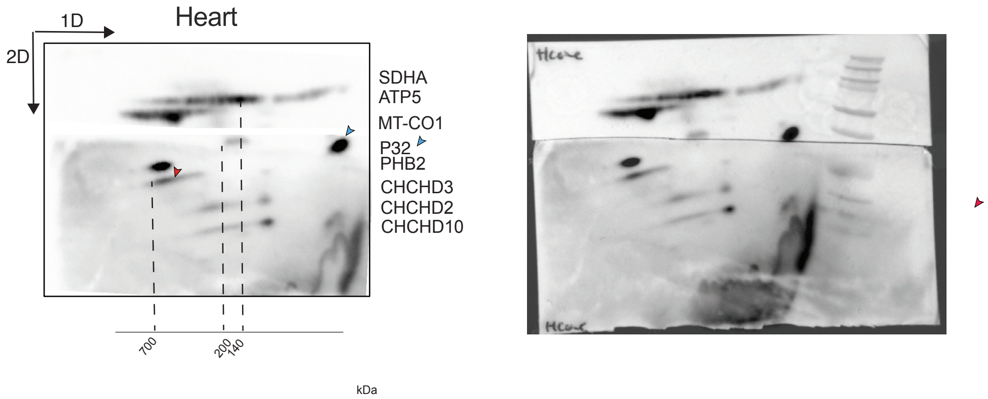

Original WB images Fig. 6D

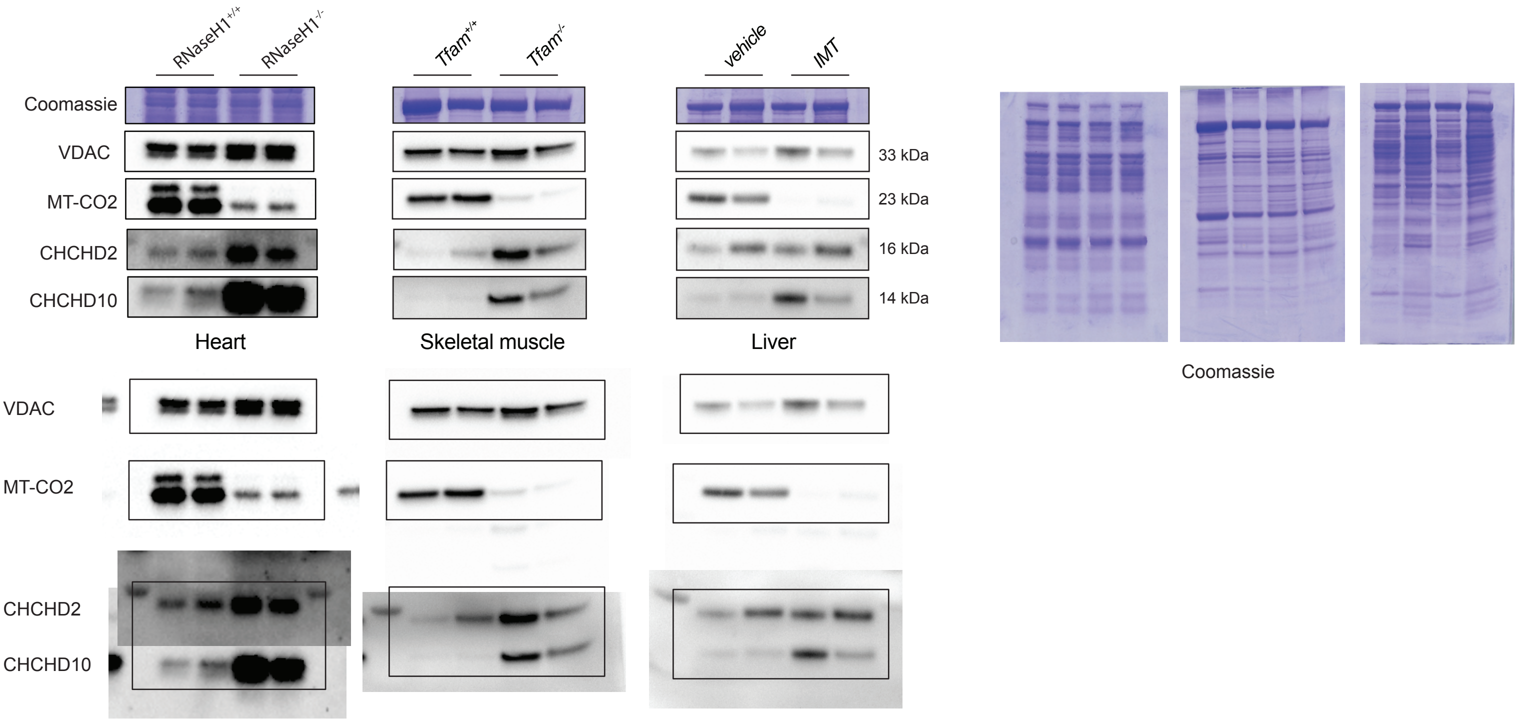

Original WB images Fig. 6F

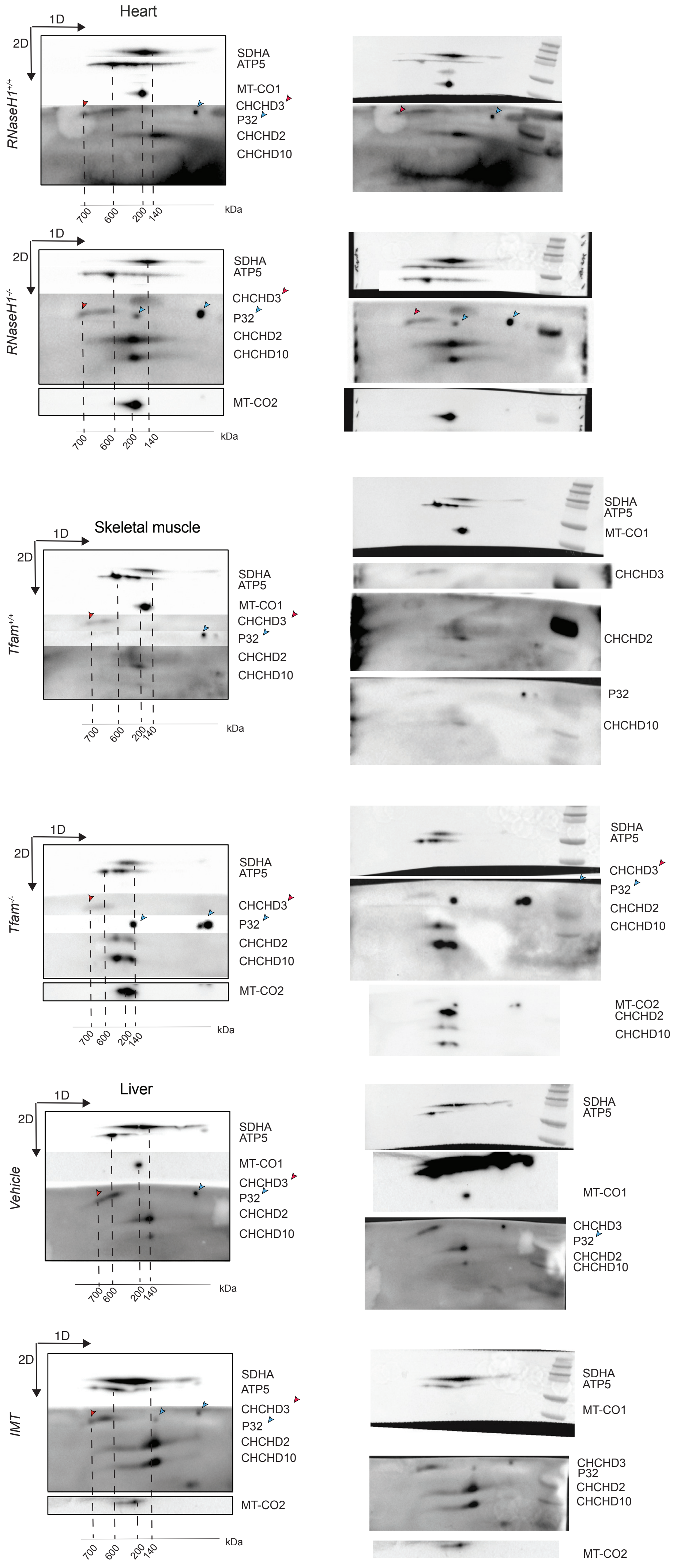

Original WB images Fig. S4D

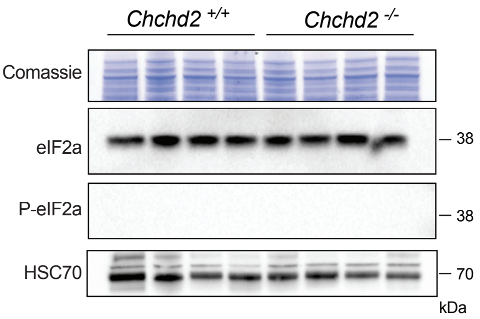

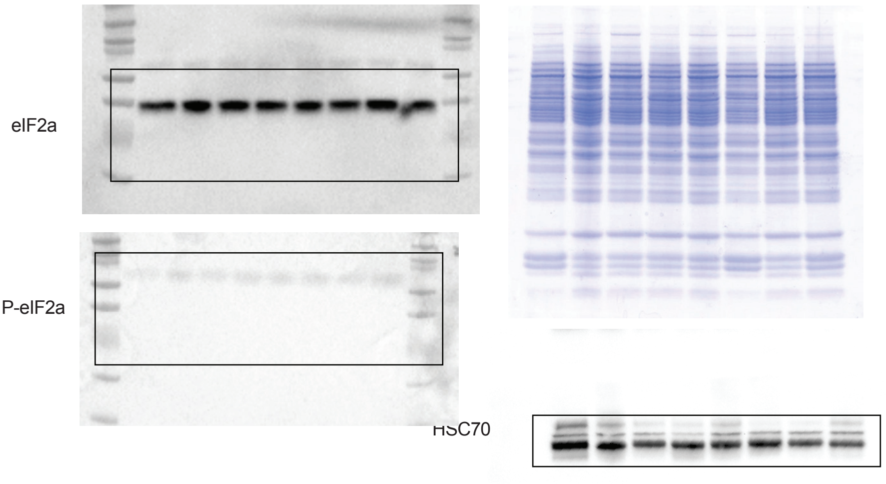

Original WB images Fig. 5A

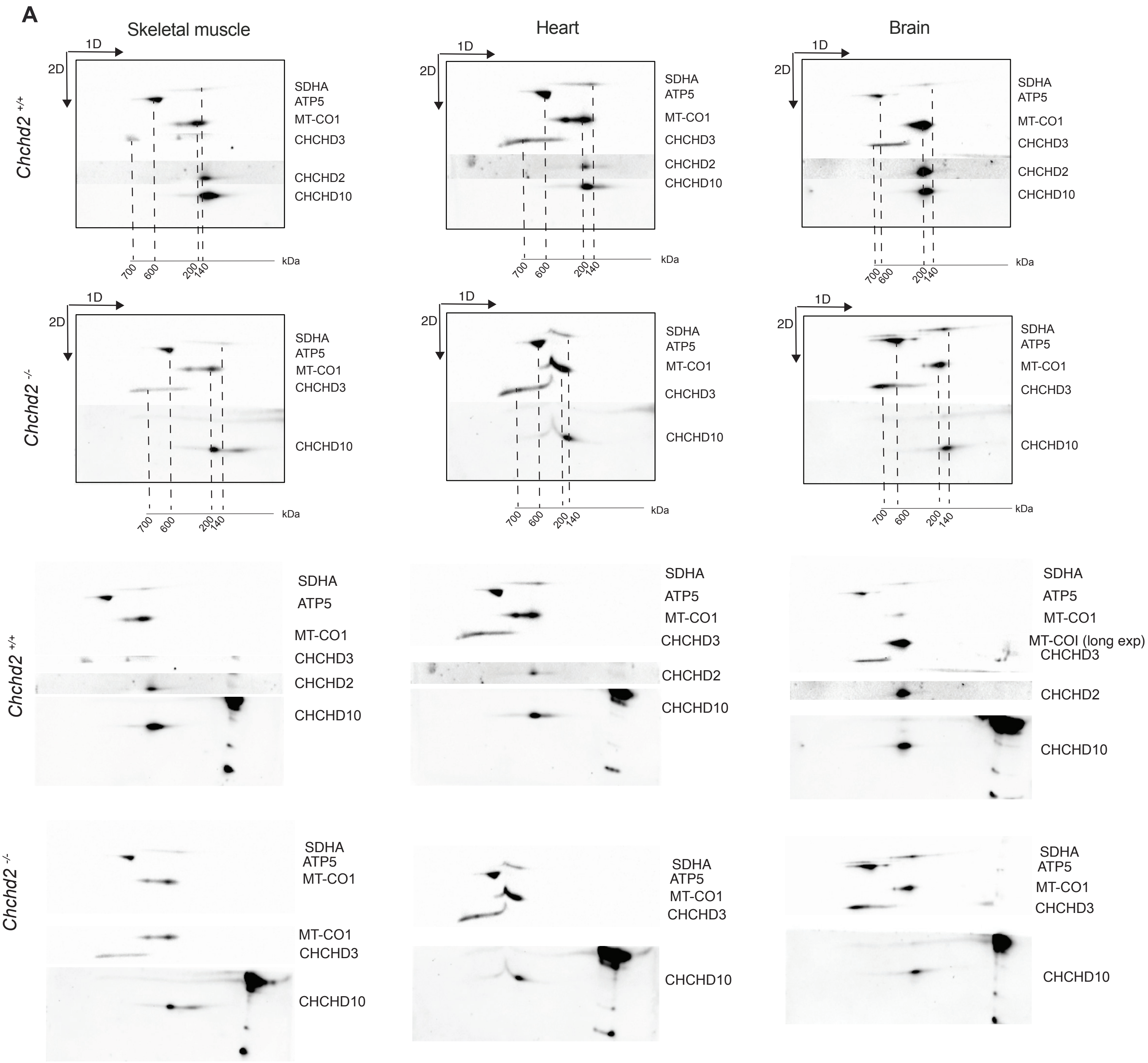

Original WB images Fig. S7A,C

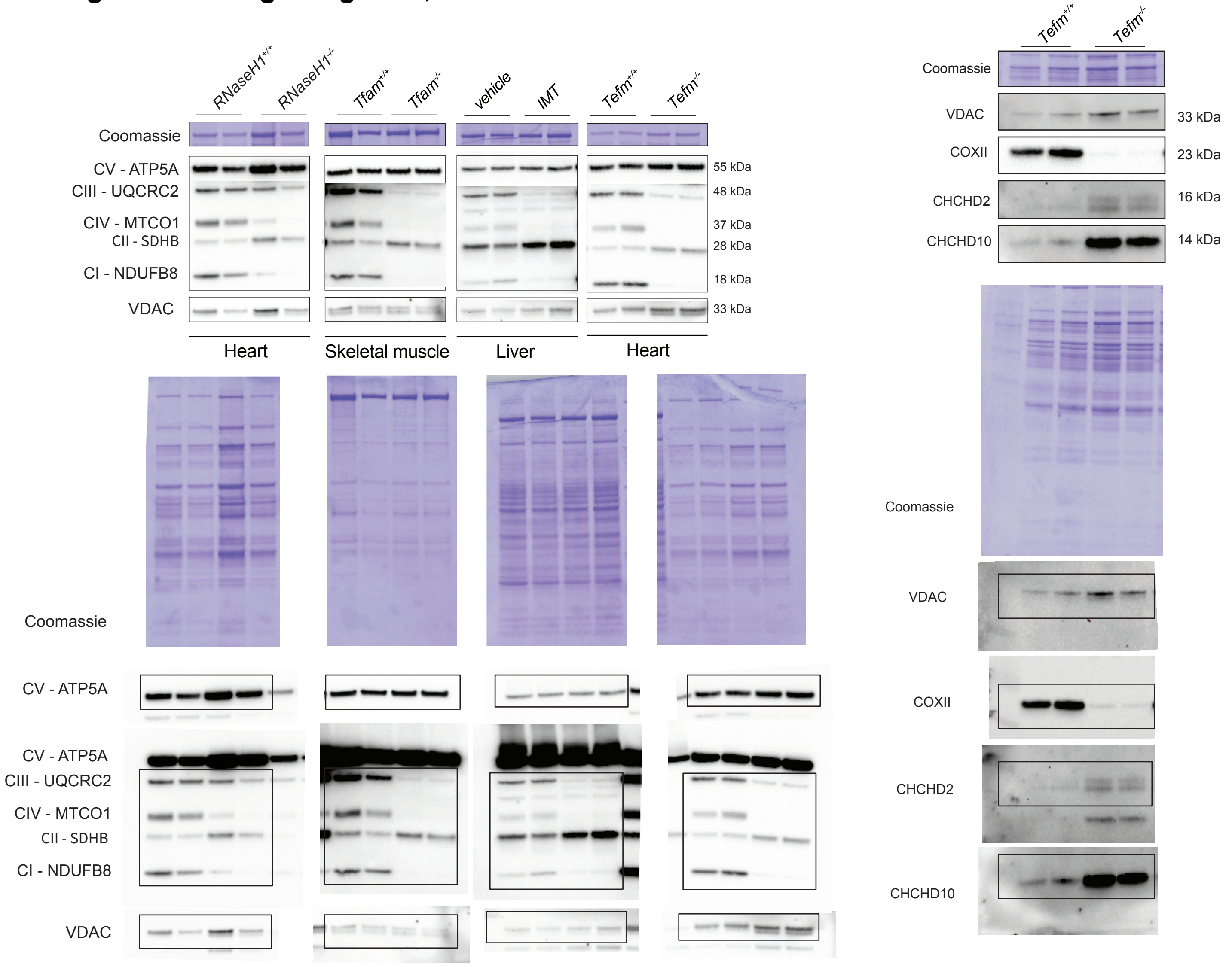

Original WB images Fig. 7F

Heart

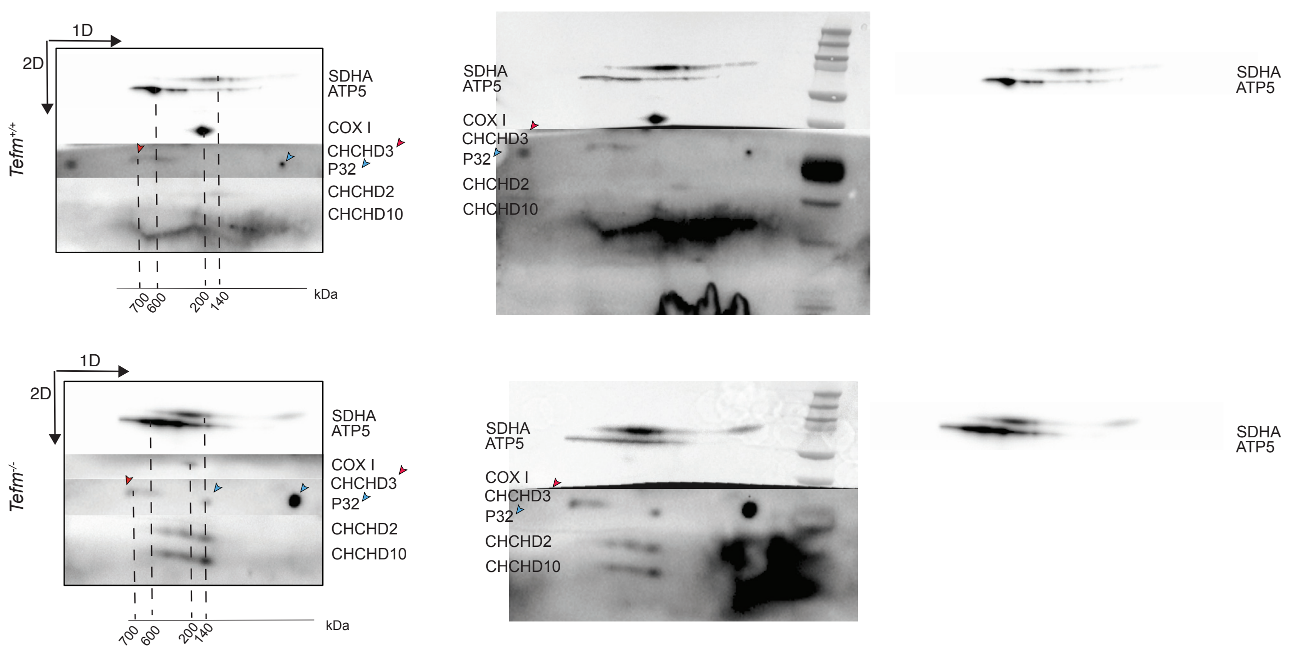

Original WB images Fig. 7A, B

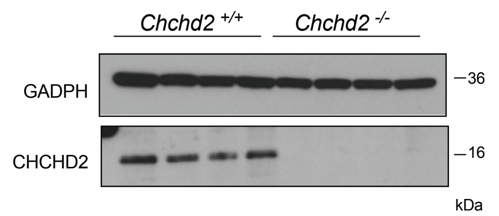

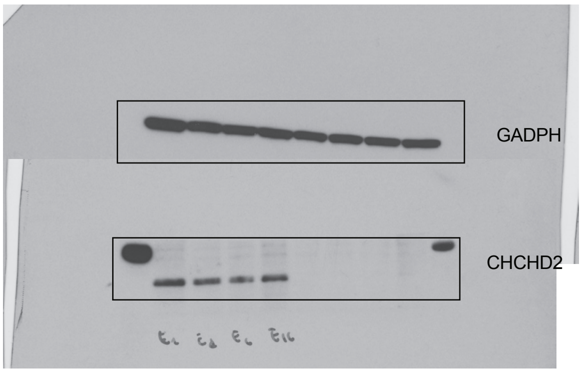

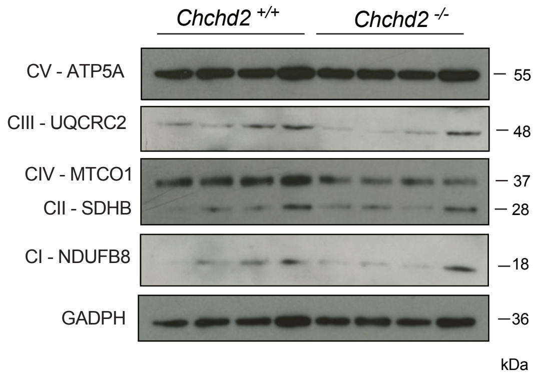

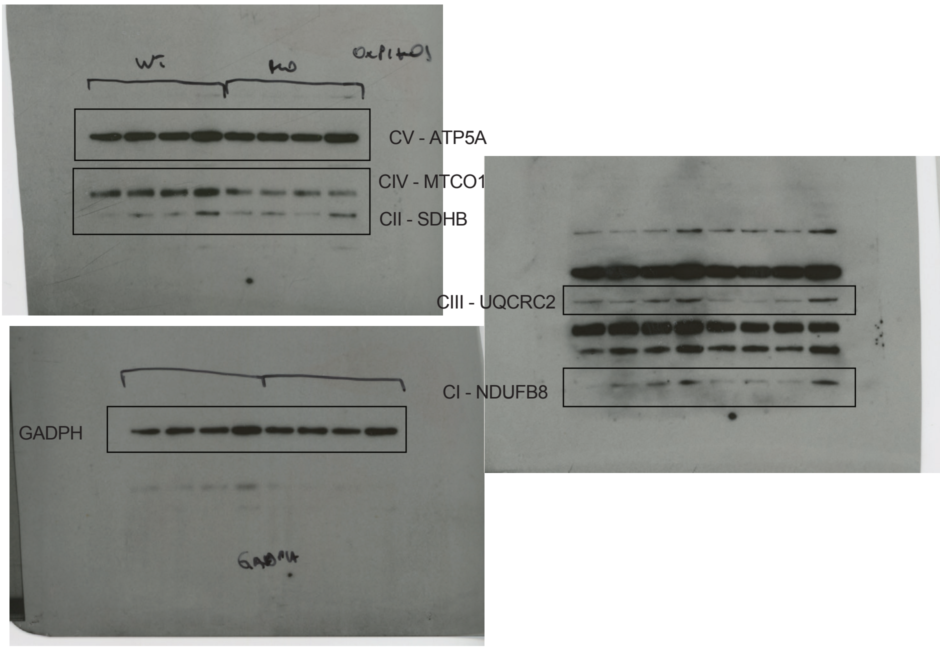

Original WB images Fig. 7D

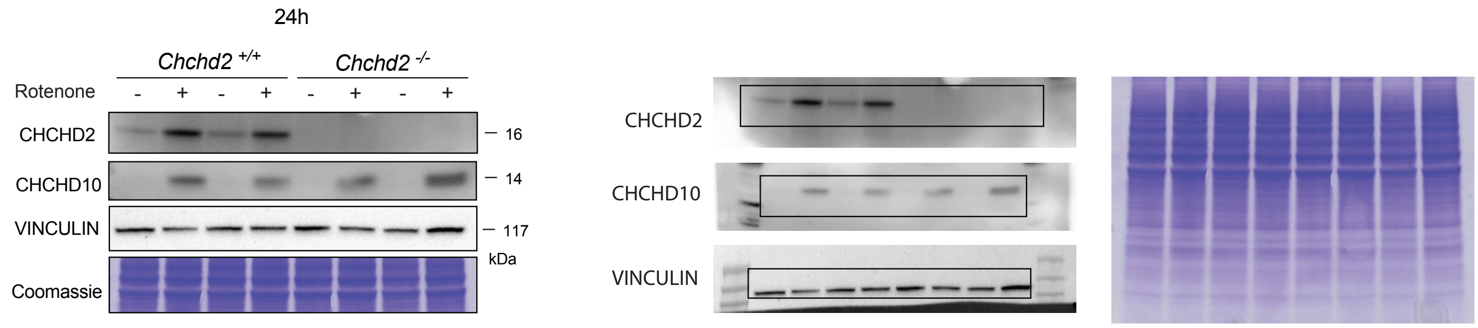

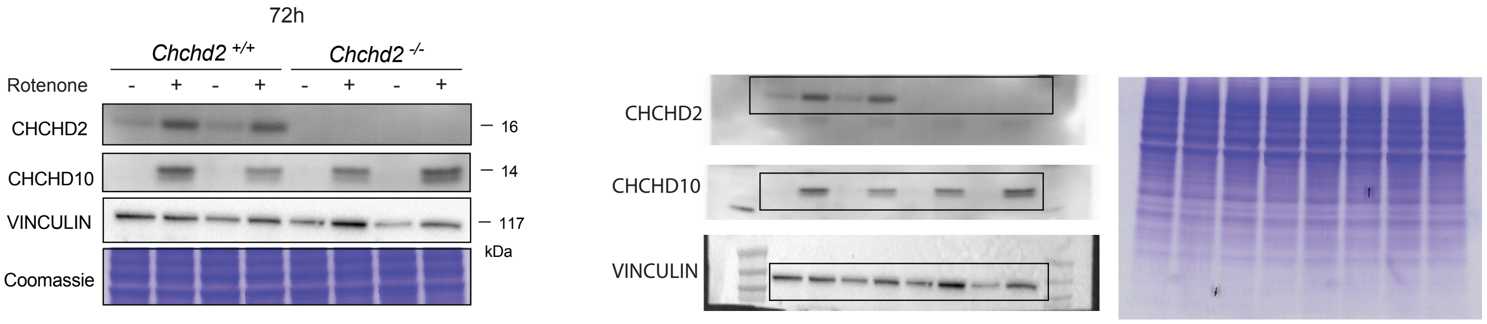

Original WB images Fig. 7E

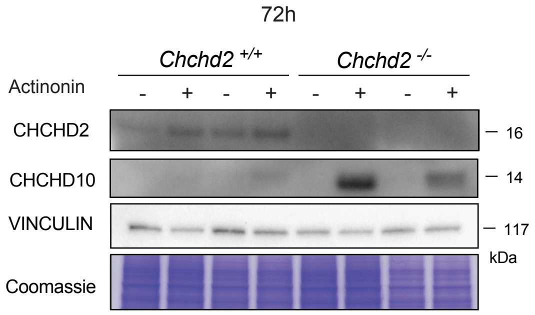

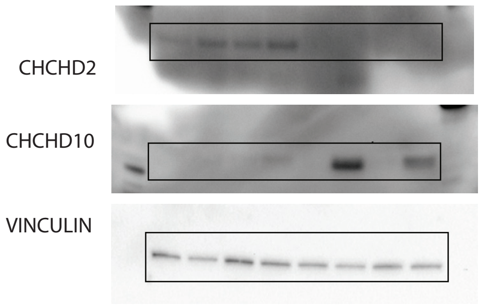

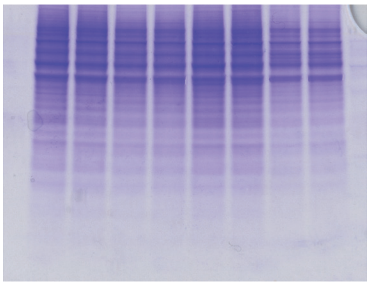

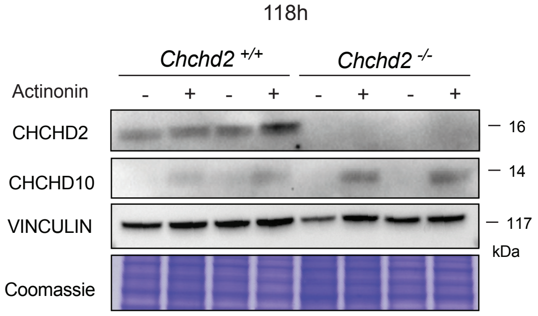

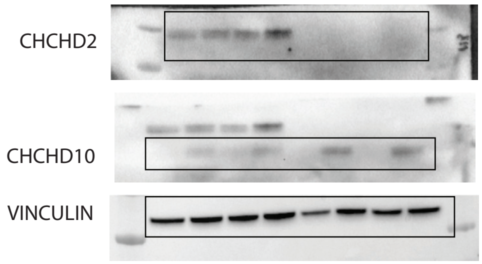

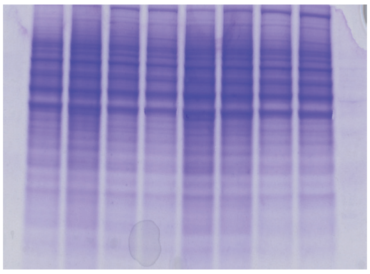

Original WB images Fig. S8A-C

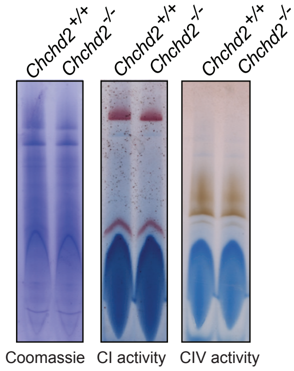

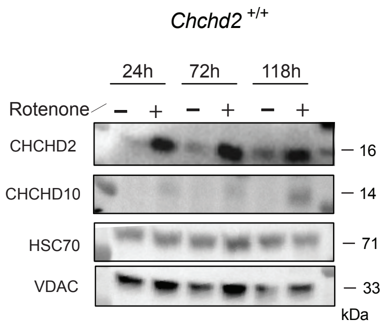

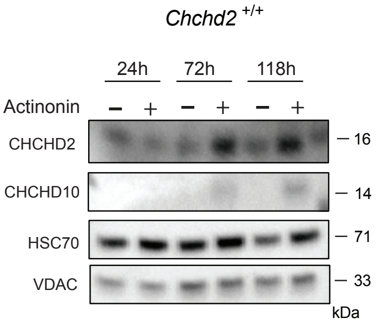

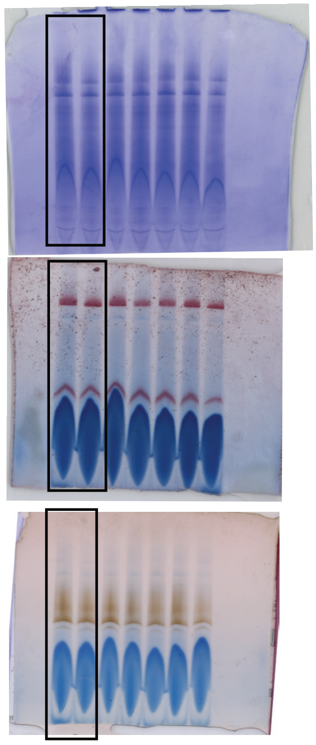

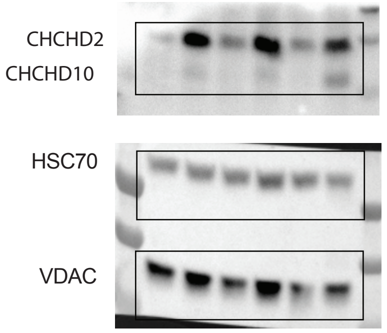

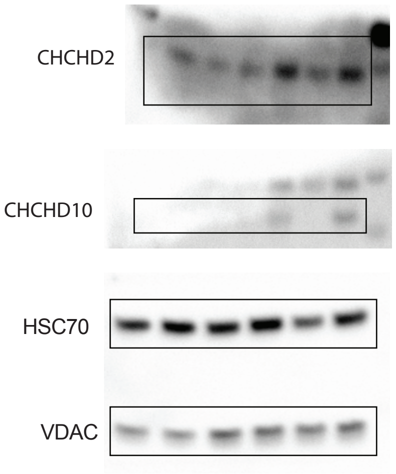

Supplement: Supplementary file 3 — Uncropped western blots [file 41419_2025_8030_MOESM3_ESM.pdf]
